# Influence of Various Processing Parameters on the Microbial Community Dynamics, Metabolomic Profiles, and Cup Quality During Wet Coffee Processing

**DOI:** 10.3389/fmicb.2019.02621

**Published:** 2019-11-13

**Authors:** Sophia Jiyuan Zhang, Florac De Bruyn, Vasileios Pothakos, Gonzalo F. Contreras, Zhiying Cai, Cyril Moccand, Stefan Weckx, Luc De Vuyst

**Affiliations:** ^1^Research Group of Industrial Microbiology and Food Biotechnology, Faculty of Sciences and Bioengineering Sciences, Vrije Universiteit Brussel, Brussels, Belgium; ^2^Nestlé Coffee Agriculture Service, Yunnan, China; ^3^Yunnan Institute of Tropical Crops, Kunming, China; ^4^Nestlé Research, Vers-chez-les-Blanc, Switzerland

**Keywords:** coffee bean fermentation, wet processing, *Coffea arabica*, amplicon sequencing, shotgun metagenomics, metabolomics, lactic acid bacteria

## Abstract

Post-harvest wet coffee processing is a commonly applied method to transform coffee cherries into green coffee beans through depulping or demucilaging, fermentation, washing, soaking, drying, and dehulling. Multiple processing parameters can be modified and thus influence the coffee quality (green coffee beans and cup quality). The present study aimed to explore the impacts of these parameters, including processing type (depulping or demucilaging), fermentation duration, and application of soaking, on the microbial community dynamics, metabolite compositions of processing waters (fermentation and soaking) and coffee beans, and resulting cup quality through a multiphasic approach. A large-scale wet coffee processing experiment was conducted with *Coffea arabica* var. Catimor in Yunnan (China) in duplicate. The fermentation steps presented a dynamic interaction between constant nutrient release (mainly from the cherry mucilage) into the surrounding water and active microbial activities led by lactic acid bacteria, especially *Leuconostoc* and *Lactococcus*. The microbial communities were affected by both the processing type and fermentation duration. At the same time, the endogenous coffee bean metabolism remained active at different stages along the processing, as could be seen through changes in the concentrations of carbohydrates, organic acids, and free amino acids. Among all the processing variants tested, the fermentation duration had the greatest impact on the green coffee bean compositions and the cup quality. A long fermentation duration resulted in a fruitier and more acidic cup. As an ecological alternative for the depulped processing, the demucilaged processing produced a beverage quality comparable to the depulped one. The application of soaking, however, tempered the positive fermentation effects and standardized the green coffee bean quality, regardless of the preceding processing practices applied. Lastly, the impact strength of each processing parameter would also depend on the coffee variety used and the local geographical conditions. All these findings provide a considerable margin of opportunities for future coffee research.

## Introduction

It has only been 500 years since coffee acquired its worldwide popularity (Yilmaz et al., 2017). Today, coffee has become one of the most important commercial crops, on which millions of people depend for their livelihood (Lambot et al., 2017). To transform the coffee cherries into a cup of coffee, a complex chain of post-harvest events is needed, during which the cherries are first picked from the coffee trees, processed to green coffee beans, subsequently roasted, and finally brewed to generate a coffee beverage with pleasant aroma and taste (Batista and Chalfoun, 2014; Brando and Brando, 2014; Silva, 2014; Poisson et al., 2017; Waters et al., 2017). Each step of this processing chain plays a significant role in the coffee quality, which can be evaluated by the quality of the green coffee beans as well as the sensory experience of the brewed coffees (i.e., cup quality) (Batista and Chalfoun, 2014). Hence, post-harvest processing offers a margin for improvement of the coffee quality.

Whereas many recent studies have investigated mainly the roasting (Bustos-Vanegas et al., 2018; Gloess et al., 2018) and brewing steps of coffee production (Parenti et al., 2014; Labbe et al., 2016), the impacts of the post-harvest processing of the coffee cherries and beans, besides genetic attributes of coffee varieties and farming practices, on the coffee quality still remain an open field of research (Lee et al., 2015; Vaughan et al., 2015; De Bruyn et al., 2017; Waters et al., 2017; Pereira et al., 2019; Zhang et al., 2019). Among numerous post-harvest processing practices, wet processing is commonly applied for *Coffea arabica* to generate high-quality Arabica coffee. During wet processing, harvested mature coffee cherries are first depulped (i.e., squeezed mechanically to remove the skin and pulp) and then fermented underwater until the mucilage (a carbohydrate-rich layer) is removed, which usually takes 12–72 h (Brando and Brando, 2014; Silva, 2014). After washing the fermented beans, they are dried until their moisture content is below 12% (m/m), and finally dehulled to yield the green coffee beans. The duration of the fermentation step has an impact on the microbial activities that take place and, hence, on the chemical composition of the green coffee beans and the resulting cup quality (De Bruyn et al., 2017; Zhang et al., 2019). Sometimes, an extra soaking step, during which the washed beans are submerged in clean water, is applied to improve the visual appearance of the green coffee beans and to obtain a clean taste in the final cup (Murthy and Naidu, 2012). Consequently, the wet processing method is time-demanding and resource-intensive and requires a high water usage and extra treatments of the fermentation and washing waters, due to their high contents of organic pollutants (Bonilla-Hermosa et al., 2014). An alternative method is to use a demucilager, a machine that can scrape off the mucilage from the depulped beans mechanically (Murthy and Naidu, 2012; Brando and Brando, 2014). In this case, the demucilaged beans are usually dried either immediately or after a short fermentation step (usually 12 h; Sanz-Uribe et al., 2017). With the help of a demucilager, the water usage and the processing time can be reduced (Chapagain and Hoekstra, 2007). Further, the waste products are more easily processed and discarded (Murthy and Naidu, 2012; Borém et al., 2014). However, the added values of demucilaging and soaking are controversial, since their impacts on the green coffee beans and cup quality are still unclear, as they have never been studied in great detail.

On top of possible variations of the processing parameters, wet processing is an interplay of microbial activities and endogenous bean metabolism, especially at the stage of fermentation (Kramer et al., 2010; Lee et al., 2015; De Bruyn et al., 2017; Waters et al., 2017; Zhang et al., 2019). Bacteria [especially lactic acid bacteria (LAB) and enterobacteria, but also acetic acid bacteria (AAB) and bacilli] and yeasts, which originate from the environment (cherry surfaces, plantation surroundings, and/or processing equipment) and are highly variable and difficult to predict, are prevalent during the fermentation step (Silva, 2014; Evangelista et al., 2015; Lee et al., 2015; Vaughan et al., 2015; De Bruyn et al., 2017; Pereira et al., 2019; Zhang et al., 2019). Their metabolites can accumulate onto the coffee beans, resulting in an indirect impact on the cup quality. As intermediate seeds, the coffee beans remain active throughout the post-harvest processing chain and respond to various abiotic stress factors, such as hypoxia during fermentation and drought stress during drying (Selmar et al., 2006; Kramer et al., 2010; De Bruyn et al., 2017; Zhang et al., 2019). Detailed monitoring of the microbial community dynamics and diversity as well as the metabolomics of both pulp and beans along the whole post-harvest processing chain has shown that on-plantation practices and fermentation under carefully controlled conditions regarding the ripeness stage of the coffee cherries and the fermentation duration selects for specific LAB species (De Bruyn et al., 2017; Zhang et al., 2019). Moreover, a long fermentation duration results in not only a microbial shift (from leuconostocs to acid-tolerant lactobacilli) but also chemical compositional changes in the green coffee beans and distinct sensory attributes in the brewed cup (De Bruyn et al., 2017; Zhang et al., 2019). As the latter studies were performed with *C. arabica* var. Typica in Ecuador, it was valuable to verify if the same effects would be obtained with a different coffee variety in a distinct geographical region. Furthermore, there are no scientific data available about how demucilaging affects the microbial ecology or metabolite compositions of beans during fermentation, which could be a compelling way of modulating the coffee flavor by inferring specific changes in the microbial consortium during fermentation.

The present study aimed to investigate the impacts of multiple processing parameters (demucilaging and depulping, fermentation duration, and application of soaking) on the dynamics of the microbial communities, the metabolomic profiles of the coffee beans and processing waters, the metabolite compositions of the green coffee beans, and the sensory quality of the coffee brews upon post-harvest processing of *C. arabica* var. Catimor cherries and beans in China.

## Materials and Methods

### Wet Processing Field Experiments

**FIGURE 1:**
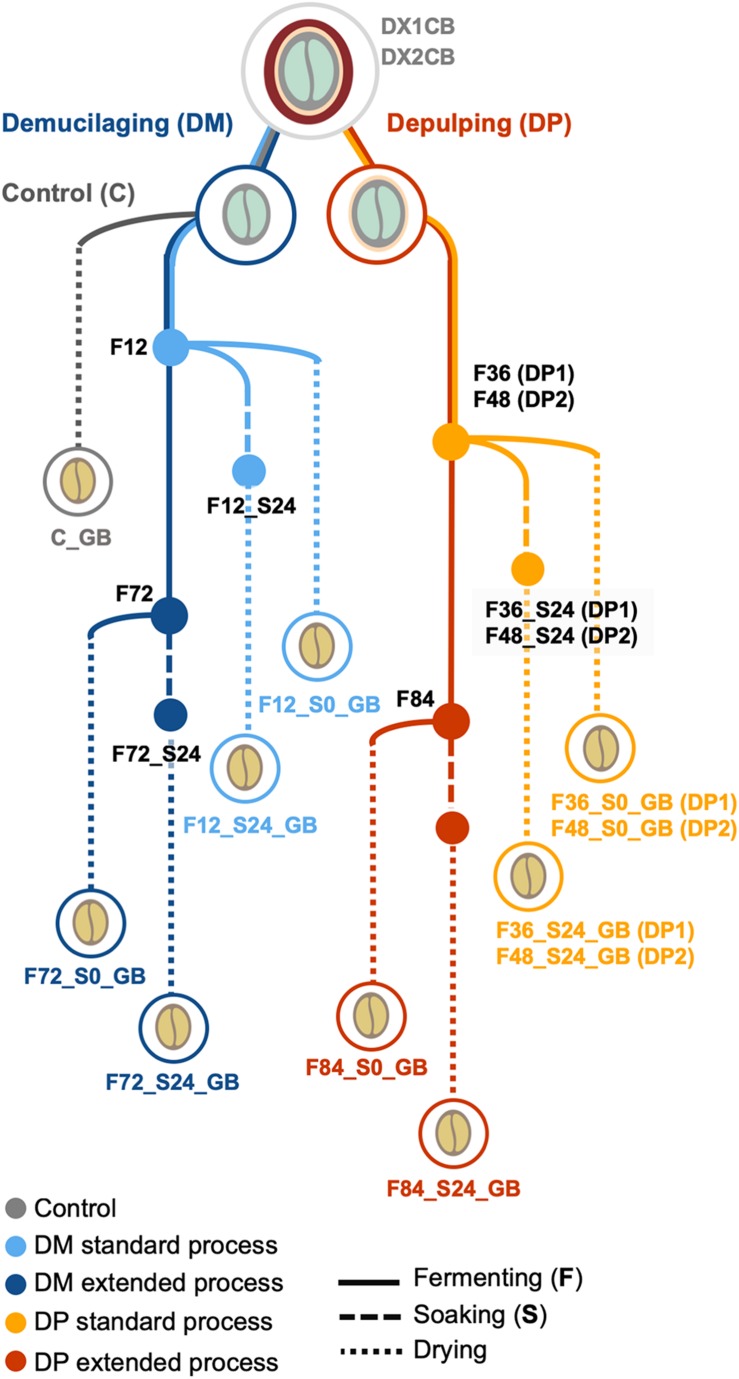
Overview of the Arabica coffee wet processing experiments, with multiple variations in processing practices, including processing type (demucilaged processing and depulped processing, referred to as DM and DP processes, respectively), fermentation duration (standard and extended), and application of soaking (with and without soaking; S0 and S24, respectively). Control processes (C), without fermentation or soaking, were also conducted. The experiments were conducted in duplicate, indicated as first (1) and second (2) trials. DX1CB and DX2CB, fresh coffee fruits; GB, green coffee beans.

Wet processing field experiments were carried out at a coffee plantation of the Experimental and Demonstration farm near Jinghong in Yunnan, China (latitude and longitude coordinates 21°58′57′′N and 101°7′23′′E, respectively; altitude, 1,300 m) in December 2015 to January 2016. During this period, cold weather conditions occurred and the daily coffee harvesting yield was 4000–5000 kg of matured coffee cherries per day. Two biological replicates were performed (1 week apart), further referred to as the first and second processing trials. Each replicate included two processing types, namely demucilaged processing (further referred to as DM1 and DM2 processes) and depulped processing (DP1 and DP2 processes) (Figure 1). For each replicate, 2000 kg of mature cherries of *C. arabica* var. Catimor were handpicked by local farmers and split evenly for the DM and DP processes. In the DP processes, the cherries were depulped with a mechanical depulper (UCBE 500; Penagos, Bucaramanga, Colombia) and the beans were subsequently submerged in clean water to ferment in a concrete tank (4.0 m × 2.5 m × 2.5 m). Removal of the mucilage determined the standard fermentation time (36 h for DP1 and 48 h for DP2). When the mucilage was removed, half of the beans were withdrawn, representing a standard fermentation, while the other half remained in the tank until 84 h of fermentation, representing an extended fermentation. In the DM processes, the cherries were depulped and demucilaged by the same machine used for the DP processes. The mucilage-free beans were then submerged in clean water to ferment in a concrete tank (same dimensions as those mentioned above). Half of the beans were withdrawn after a fixed duration of 12 h (according to local practices), representing a standard fermentation, while the other half remained in the tank until 72 h of fermentation, representing an extended fermentation. After all fermentations, the beans were washed thoroughly by passing them through a curved water channel. Part of the washed beans (approximately 125 kg) were dried immediately, while the rest of the beans (approximately 875 kg) were placed in a plastic bucket and soaked for 24 h in clean water before drying. All beans were dried on a concrete patio with frequent mixing until the moisture content was below 12% (m/m). Together with each replicate, a negative control was implemented, for which the demucilaged beans (approximately 125 kg) were dried immediately, without fermentation or soaking, further referred to as control processes (C).

The temperature and pH of the fermenting masses were monitored on-line by means of a WTW pH 3110 data logger (Xylem Analytics, Weilheim, Germany). For off-line analyses, multiple samples were taken along the processing duration, encompassing fresh coffee cherries (DX1CB for the first trial and DX2CB for the second trial), beans and fermentation waters during the fermentation step (FB and FW, respectively), beans and soaking waters during the soaking step (SB and SW, respectively), and the final green coffee beans (GB). Microbiological plating for culture-dependent analysis was performed on-site. The bean and processing water samples were frozen immediately at −20°C for culture-independent and metabolomic analysis after shipping to Belgium. The GB of each processing variant were shipped to Nestlé Research (NR, Vers-chez-les-Blanc, Switzerland) at room temperature and used there for coffee brewing and sensory analysis.

In an additional experiment, a prolonged submersion of beans and pulps was conducted to investigate the potential diffusion of coffee substrates into the surrounding waters (further referred to as bean water and pulp water). Freshly demucilaged beans and pulp were collected from a same harvest of coffee cherries (80 kg). The demucilaged beans were thoroughly rinsed and any pulp present was removed manually. Subsequently, the pulp-free demucilaged beans (B) and the fresh pulps from the depulper (P) were submersed separately in buckets with clean water for 72 h. The bean and pulp waters, beans, and pulps at the beginning and the end of submersion were sampled and frozen immediately at −20°C for metabolomic analysis after shipping to Belgium. The beginning and the end of submersion were denoted as D0 and D72.

### Culture-Dependent Microbiological Analysis

Microbial counts were monitored by plating on selective agar media, as described previously (Zhang et al., 2019). The total aerobic microbiota was targeted on plate count agar (PCA; Oxoid, Basingstoke, Hampshire, United Kingdom), LAB were targeted on de Man-Rogosa-Sharpe agar supplemented with sorbic acid (1%, m/v) (MRS-S agar; Pothakos et al., 2014), AAB on modified deoxycholate-mannitol-sorbitol agar (mDMS agar; Papalexandratou et al., 2013), and yeasts and filamentous fungi on yeast-glucose agar (YG agar; Laureys and De Vuyst, 2014). Enterobacteria were targeted by plating on Rapid’*Enterobacteriaceae* agar (BioRad Laboratories; Hercules, CA, United States). Counts are represented as triplicate averages with their standard deviations. Isolate recovery (10–15 colonies per agar medium and per time point corresponding with the highest dilutions) and dereplication and identification were performed for LAB, yeasts, and AAB, as described previously (Zhang et al., 2019). Concisely, genomic DNA of pure cultures was extracted and specific loci were sequenced to allocate species level identity for each isolate. LAB were identified by sequencing the 16S rRNA gene, AAB by sequencing the 16S rRNA gene and *dnaK* gene, and yeasts by sequencing the internal transcribed spacer (ITS) region of the fungal ribosomal RNA transcribed unit. The accession numbers of the reference sequences used for identification of the isolates are represented in Table 1.

**TABLE 1:** Accession numbers of the reference sequences used for species level identification of the isolates obtained from the Arabica coffee post-harvest processing experiments.

**Species identification**	**Accession number (NCBI nucleotide database)**
**Yeasts**	**ITS sequences**
*Candida humilis*	JQ726600
*Candida quercitrusa*	KF728800
*Candida solani*	KY102402
*Cordyceps brongniartii*	JN941759
*Hanseniaspora uvarum*	KJ706285
*Hanseniaspora vineae*	HQ909094
*Lachancea lanzarotensis*	KY076618
*Papiliotrema terrestris*	KY104479
*Pichia kluyveri*	LT714692
*Saccharomyces cerevisiae*	KJ781352
*Starmerella bacillaris*	KY076623
*Torulaspora delbrueckii*	KM384545
*Wickerhamomyces anomalus*	KC597821
**Lactic acid bacteria**	**16S rRNA gene sequences**
*Lactobacillus coryniformis*	EU626008
*Lactobacillus plantarum*	LT593850
*Lactococcus hircilactis*	NR_136465
*Lactococcus lactis*	MF429271
*Leuconostoc citreum*	NR_041727
*Leuconostoc holzapfelii*	NR_042620
*Leuconostoc mesenteroides*	KC108669
*Leuconostoc pseudomesenteroides*	GQ351323
*Weissella soli*	NR_025642

*NCBI, National Center for Biotechnology Information; ITS, internal transcribed spacer.*

### Culture-Independent Microbiological Analysis

#### Metagenetics

Total microbial DNA extraction and amplicon sequencing were done as described previously (Zhang et al., 2019). Concisely, total microbial DNA was extracted from each sample, based on multiple cell lysis steps (targeting bacteria and fungi), phenol/chloroform/isoamyl alcohol extraction, and on-column purification. The V4 hypervariable region of the bacterial 16S rRNA gene and the ITS1 region of the fungal 26S rRNA gene were amplified selectively. These amplicons were sequenced on the MiSeq platform (Illumina; San Diego, CA, United States) of the Brussels Interuniversity Genomics High Throughput core facility BRIGHTcore (Jette, Belgium). For the bioinformatic analysis, the resulting sequences were converted into relative abundances of amplicon sequence variants (ASVs) with the R-package DADA2 (Callahan et al., 2017). The sequences are available at the European Nucleotide Archive under accession numbers PRJEB30537 for the V4 sequences^1^ and PRJEB30538 for the ITS1 sequences^2^.

#### Shotgun Metagenomics

##### Shotgun metagenomic sequencing and quality processing

Shotgun metagenomic sequencing was applied on six well-chosen underwater fermentation samples, namely those corresponding with fermentation time point 72 h of both DM processes (DM1_F72 and DM2_F72), and the time points that represented the end of the standard fermentation duration for the two DP processes (DP1_F36 and DP2_F48) and the end of the extended fermentation duration for the DP processes (DP1_F84 and DP2_F84). A DNA-based metagenomic analysis was chosen to perform both a taxonomic assignment of metagenomic reads and a functional analysis based on contigs of the same metagenomic reads. Hereto, the total microbial DNA, extracted as described above, was fragmented into pieces with an average size of 550 base pairs (bp), using a Covaris M220 device (Covaris, Brighton, United Kingdom). Barcoded libraries were created using a KAPA Hyper Prep Kit (KAPA Biosystems, Wilmington, MA, United States), according to the manufacturer’s instructions. After size selection using Agencourt AMPure XP magnetic beads (Beckman Coulter, Brea, IN, United States), the size of the fragments in the final libraries was controlled using an Agilent 2100 Bioanalyzer (Agilent Technologies, Santa Clara, CA, United States). The concentrations were measured using a fluorescence-based method (Qubit 2.0; Thermo Fisher Scientific, Wilmington, DE, United States). All libraries with a concentration above 1.0 ng/μL and a concordant size range were quantified based on qPCR using a LightCycler 480 II (Roche Diagnostics, Basel, Switzerland) and the KAPA Illumina Library Quantification Kit (KAPA Biosystems). Subsequently, all libraries were diluted to a concentration of 2 nM. Per sequencing run, three libraries were pooled equimolarly prior to denaturation with NaOH, followed by a dilution to 7 pM and final paired-end (PE) sequencing by means of a MiSeq sequencer (Illumina) and MiSeq Reagent Kit v3 (600-Cycle). Library preparation and sequencing were performed by BRIGHTcore. The sequence data obtained was demultiplexed and quality-processed using Trimmomatic-0.36 (Bolger et al., 2014). Only the paired forward and reverse metagenomic sequence reads were retained, which were merged into contigs, further referred to as metagenomic sequences, using PANDAseq v2.7 (Masella et al., 2012) with the minimum overlap set to 10 nucleotides (nt).

To remove metagenomic sequences that were derived from the coffee plant, the metagenomic sequences were aligned to the genome sequence of *Coffea canephora* (RefSeq accession number PRJEB4211). Although the experiments were performed using *C. arabica*, an allotetraploid plant and a hybrid between the diploid *C. canephora* and *Coffea eugenoides*, its genome was not publicly available at the time of analysis. Alignment was performed using the BLAST algorithm blastn (Altschul et al., 1990) with the word size set to 25 nt, the minimum alignment identity to 80%, the maximum alignment hits per read to 1, and the query coverage to 70%.

##### Taxonomic assignment of metagenomic reads

For taxonomic assignment, the metagenomic reads were aligned using the blastn algorithm, with the same settings as mentioned above, to a customized database containing all available genomes of those genera that were present during coffee processing, a strategy also known as metagenomic recruitment plotting (Vermote et al., 2018). Hereto, a total of 561 genome sequences were downloaded from the National Center for Biotechnology Information (NCBI) RefSeq and Whole Genome Shotgun (WGS) databases, comprising genome sequences from *Acetobacteraceae* (*n* = 44), *Lactobacillaceae* (*n* = 175), *Leuconostocaceae* (*n* = 35), *Enterococcaceae* (*n* = 26), *Streptococcaceae* (*n* = 32), *Enterobacterales* (*n* = 99), fungi (*n* = 56), and miscellanea (*n* = 94). Genera that were represented by less than 0.1% of all metagenomic reads were not further considered.

##### Functional analysis

Assembly of the metagenomic reads into contigs was done using the MEGAHIT assembler (Li et al., 2015), with the pre-set parameter set to “meta sensitive” for each time point independently. Only contigs with a length higher than 1 kbp were retained. The metagenomic contigs assembled were annotated with Prokka (Seemann, 2014). The annotations obtained were filtered for genes encoding known proteins and designated with an EC number, reflecting the reactions catalyzed by the enzymes encoded. Subsequently, per sample, the occurrence of each EC number was quantified, and the list of EC numbers was condensed to the sub-subclass level.

The metagenomic shotgun reads are available at the European Nucleotide Archive under accession number PRJEB31746^3^.

### Meta-Metabolomic Analysis

#### Sample Preparation

The fermentation and soaking water samples were first thawed and then microcentrifuged (19,400 × *g* for 15 min at 10*°*C) to remove the remaining plant material. The clear supernatants were collected and subjected to a metabolomic analysis. In the case of the bean samples taken at the fermentation and soaking steps and the GB, the parchment and silver skin were removed manually. The resulting beans were then frozen in liquid nitrogen and ground to fine powders for extraction. Three different extraction procedures were performed on the bean powders (in triplicate), namely aqueous [ultrapure water (Milli-Q; Merck, Billerica, MA, United States)], acidic (0.01 N HCl; Merck), and organic solvent (40%, v/v, methanol; Merck) extraction, during which ethylenediaminetetraacetic acid (0.2%, m/m, Merck) and ascorbic acid (0.2%, m/m, Merck) were added to inhibit enzyme activity and oxidation, respectively. The moisture content of the beans was determined in triplicate by means of an oven method, during which the ground bean powders were dried at 105*°*C for 24 h. All concentrations of bean metabolites are expressed on a dry mass basis, unless stated otherwise.

#### Quantification of Simple Carbohydrates and Sugar Alcohols

The concentrations of simple carbohydrates (fructose, galactose, glucose, mannose, and sucrose) and sugar alcohols (arabitol, erythritol, glycerol, mannitol, myo-inositol, sorbitol, and xylitol) in the processing waters and aqueous bean extracts were determined by high-performance anion exchange chromatography with pulsed amperometric detection (HPAEC-PAD), using an ICS 3000 chromatograph equipped with a CarboPac PA-100 and CarboPac MA-1 column (Dionex, Sunnyvale, CA, United States), respectively, as described previously (Zhang et al., 2019). Quantification was performed via internal standardization in triplicate. The internal standard (IS) solution consisted of rhamnose (20 mg/L; Merck, Darmstadt, Germany) in acetonitrile (Merck). All samples were mixed with the IS solution, microcentrifuged (19,400 × *g* for 15 min at 10°C), and filtered [Chromafil 0.20 μm polytetrafluoroethylene (PTFE; in the case of simple carbohydrates) filters or polyethersulfone filters (in the case of sugar alcohols); Macherey-Nagel, Düren, Germany] before injection (10 μL) into the column.

#### Quantification of Organic Acids

The concentrations of organic acids (i.e., citric acid, fumaric acid, gluconic acid, isocitric acid, lactic acid, malic acid, oxalic acid, quinic acid, and succinic acid) in the processing waters and acidic bean extracts were determined by ultra-performance liquid chromatography coupled to tandem mass spectrometry (UPLC-MS/MS), using an Acquity UPLC system equipped with an HSS T3 column and a TQ tandem mass spectrometer (Waters; Milford, MA, United States), as described previously (Zhang et al., 2019). Quantification was performed through external calibration in triplicate. All samples were mixed with methanol, microcentrifuged (19,400 × *g* for 15 min at 10°C), and filtered (Chromafil 0.2 μm PTFE filters) before injection (10 μL) into the column.

#### Quantification of Chlorogenic Acids

The concentrations of six chlorogenic acid (CGA) isomers, namely 3-caffeoylquinic acid (CQA), 4-CQA, 5-CQA, 3,4-diCQA, 3,5-diCQA, and 4,5-diCQA, in the processing waters and methanol bean extracts were determined by UPLC-MS/MS, as described previously (Zhang et al., 2019). Quantification was performed through internal standardization in triplicate. The IS solution consisted of rosmarinic acid (1.0 mg/L; Merck) in acetonitrile (Merck). All samples were mixed with the IS solution, microcentrifuged (19,400 × *g* for 15 min at 10°C), and filtered (Chromafil 0.2 μm PTFE filters) before injection (10 μL) into the column.

#### Quantification of Alkaloids and Other Phenolic Acids

The concentrations of alkaloids (i.e., caffeine and trigonelline), ferulic acid, and caffeic acid in the processing waters and acidic bean extracts were determined by UPLC-MS/MS, as described previously (Zhang et al., 2019). Quantification was performed through internal standardization in triplicate. The IS solution consisted of 1-ethyl-4-(methoxycarbonyl)pyridinium iodide (0.15 mg/L; Merck) in acetonitrile (Merck). All samples were mixed with the IS solution, microcentrifuged (19,400 × *g* for 15 min at 10°C), and filtered (Chromafil 0.2 μm PTFE filters) before injection (10 μL) into the column.

#### Quantification of Free Amino Acids

The concentrations of 20 proteinogenic amino acids and one non-proteinogenic amino acid (γ-aminobutyric acid, GABA) in the processing waters and acidic bean extracts were determined by HPLC coupled to tandem MS (HPLC-MS/MS) by means of an Alliance 2695 chromatograph and Micromass Quattro Micro^TM^ mass spectrometer (Waters), as described previously (Zhang et al., 2019). Quantification was performed through internal standardization in triplicate. L-2-amino butyric acid (1.2 ng/mL; Merck) was used as IS. All samples were microcentrifuged (19,400 × *g* for 15 min at 10°C) and filtered (Chromafil 0.2 μm PTFE filters) before injection (10 μL) into the column.

#### Quantification of Short-Chain Fatty Acids and Low-Molecular-Mass Volatiles

The concentrations of short-chain fatty acids (SCFAs, i.e., acetic acid, butanoic acid, hexanoic acid, 2-methylpropanoic acid, 3-methylbutanoic acid, pentanoic acid, and propanoic acid) and low-molecular-mass volatiles (i.e., acetaldehyde, ethanol, ethyl acetate, ethyl lactate, and isopentyl acetate) in the processing waters and aqueous bean extracts were determined by gas chromatography with flame ionization detection (GC-FID), as described previously (De Bruyn et al., 2017). Quantification was performed through internal standardization in triplicate. Briefly, the samples were prepared with an IS solution of 1-butanol (0.20 g/L; Merck) and injected (1 μL; split 20) into a Stabilwax-DA column (Restek, Bellefonte, PA, United States) of a Focus GC equipped with a flame ionization detector FID-80 (Interscience, Breda, Netherlands).

#### Volatile Fingerprinting of Selected Green Coffee Beans

Semi-quantitative volatile fingerprinting of the GB samples was conducted by headspace/solid-phase microextraction coupled with GC and time-of-flight MS (HS/SPME-GC-TOF-MS) in triplicate, as described previously (Zhang et al., 2019). A Trace 1300 gas chromatograph (Thermo Fisher, Waltham, MA, United States) equipped with a Stabilwax^®^-MS column (Restek) and a BenchTOF-HD mass spectrometer (Markes International, Llantrisant, Wales, United Kingdom) was used. GB powder (1.0 g) was incubated in a 10 mL screw-top headspace vial at 50°C for 10 min, followed by extraction using a SPME fiber (DVB/CAR/PDMS, 50/30 μm; Supelco, Merck) for 45 min. The raw data were deconvoluted with TOF-DS software (Markes), followed by identification of each peak via the NIST library (National Institute of Standards and Technology, Gaithersburg, MD, United States) and the Kovats Index (Afeefy et al., 2017). The peak area of each compound identified, normalized to the peak area of the IS and adjusted for the moisture content, was a measure of the aroma intensity (Zhang et al., 2019).

### Roasting and Sensory Evaluation

Bean roasting and cup evaluation were performed according to an in-house standardized protocol (NR). GB from all processing variants were roasted until the color of the roasted beans reached a color test Neuhaus (CTN) value of 90 (Neuhaus Neotec, Ganderkesee, Germany). Coffees were brewed with a Moccamaster coffee machine (Technivorm, Amerongen, Netherlands) at a ratio of 50 g of roasted beans per liter of water. They were served at 70°C in 80 mL plastic cups. A quantitative descriptive analysis was applied to measure the intensity of 27 sensory attributes, covering eleven odor attributes (OD), 13 flavor attributes (FL), and 3 texture attributes (TX). The samples were evaluated by 12 trained panelists at NR.

### Statistical Analysis

The statistical analyses were conducted and visualized by various R packages in RStudio (version 1.1.423; R Core Team, 2018), including corrplot (Wei and Simko, 2017), ggplot2 (Wickham, 2016), gplots (Wickham, 2019), Hmisc (Harrell, 2018), lme4 (Bates et al., 2015), and vegan 2.5-2 (Oksanen et al., 2018).

To explore the isolate identification data, a (-1,1)-scaled and rotated principal component analysis (PCA) was performed on a covariance matrix with and without the (-1,1)-scaled factor loadings overlaid. To identify discriminating microbial communities between the fermentations of the DM and DP processes and to quantify the effect of the fermentation duration on the microbial communities within the fermentations of these processes, as determined culture-independently, a permutational analysis of variance (PERMANOVA) based on the Bray-Curtis dissimilarity matrix (10^5^ permutations) and similarity percentage analysis were performed with the package vegan.

For further processing of the outcome of the functional analysis of the shotgun metagenomic data, a heatmap was constructed using the gplots package, with the data scaled per sub-subclass and represented by a *Z* score.

To analyze the metabolomic data, heatmaps of the fermentation and soaking water sample data were plotted, based on Z-scores, and the data were clustered based on the average distance between the points. Linear mixed effect models (LMEM) were applied on the quantitative metabolomic data of the GB, the volatile fingerprinting data of the GB, and the sensory outcomes of the brewed coffees with the package lme4. The model included three fixed effects, namely processing type (DM and DP), fermentation duration (no fermentation, standard fermentation, and extended fermentation), and application of a soaking step (with and without soaking). The biological repetition (first and second trials) was included as random effect in the model. The significant levels of each fixed effect on the compounds targeted or sensory attributes were calculated by comparing the full model with a reduced model through an analysis of variance (ANOVA), for which the targeted fixed effect was excluded. The relative size of the effect (*t*-value) of the compound or sensory attribute was reported when the p-value was < 0.05. In the case of GB volatile fingerprinting, the volatiles of significant difference were grouped together, based on their corresponding odor descriptions (Deibler and Delwiche, 2004; Mosciano, 2018). PCAs were performed on both the bean and water metabolomic datasets. Various PCAs were plotted based on the quantitative metabolomic results of the FB and SB, the quantitative metabolomic results of the GB, and the sensory outcomes of the brewed coffees. Correlation matrices were used for the metabolite compositions of the beans, whereas a covariance matrix was used for the sensory outcomes. The number of PCs retained were derived from the Scree plot and eigenvalues obtained. Correlation analyses were applied to link the metabolomic data of the bean and water samples during the fermentation and soaking steps with the underwater submersion duration, with the packages Hmisc and corrplot. Based on Spearman correlations, only the correlations with a *p*-value < 0.05 were reported. Boxplots were used to plot the metabolite compositions of beans before (during the fermentation and soaking steps) and after drying (GB).

## Results

### Temperature and pH

The temperature at the start of all fermentation variants was approximately 15°C (Supplementary Figure S1). During fermentation, the temperature followed the day-night cycle. The final temperatures of the fermentations of the DM processes were higher than those of the DP ones (approximately 13°C versus 11°C, respectively). For all fermentation variants, the initial pH was approximately 6.0–6.5 and decreased continuously until approximately 4.0. For the fermentations of both the DM and DP processes, the major pH drop happened after 36 h of fermentation.

### Microbial Load of the Harvested Coffee Cherries

**FIGURE 2:**
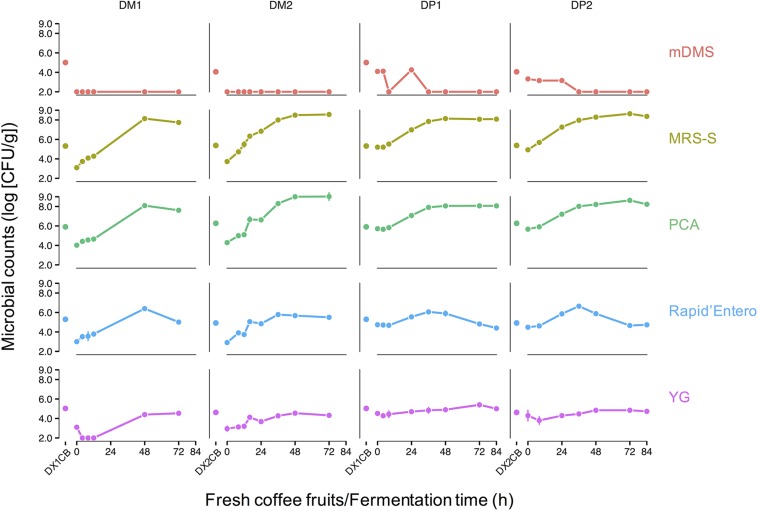
Microbial counts of fresh coffee fruits (DX1CB and DX2CB) and fermenting beans of the Arabica coffee wet processing experiments, representing fermentation of depulped (DP) and demucilaged (DM) beans, as determined through cultivation on five selective agar media, namely modified deoxycholate-mannitol-sorbitol (mDMS) agar medium for the enumeration of presumptive acetic acid bacteria, de Man-Rogosa-Sharpe agar medium supplemented with sorbic acid (MRS-S) for the enumeration of presumptive lactic acid bacteria, plate count agar (PCA) medium for the enumeration of the total aerobic microbiota, Rapid’*Enterobacteriaceae* agar medium for the enumeration of presumptive enterobacteria, and yeast-glucose (YG) agar medium for the enumeration of presumptive yeasts and filamentous fungi. Every row represents a selective agar medium and every column represents a process followed. The first disconnected time point represents the microbial load on the surfaces of the coffee fruits. The following connected time points represent the microbial counts during fermentation.

The microbial counts on the surfaces of the harvested coffee cherries ranged from log 4.0–6.0 (CFU/g) (Figure 2). The highest microbial counts were found on PCA, MRS-S agar, and Rapid’*Enterobacteriaceae* agar [approximately log 6.0 (CFU/g)], indicating a relatively high presence of aerobic microorganisms, presumptive LAB, and presumptive enterobacteria, respectively. Lower counts were found on mDMS agar and YG agar [approximately log 4.0 (CFU/g)], indicating a relatively low presence of presumptive AAB and presumptive yeasts.

### Culture-Dependent Microbial Community Dynamics and Species Diversity During Fermentation

The growth patterns of the microbial groups targeted showed no major differences between all fermentation variants of the DP and DM processes. Presumptive LAB and aerobic microorganisms were the most prevalent microbial groups in all fermentation variants. These two microbial groups displayed very similar growth patterns during fermentation. The presumptive LAB reached counts that were several orders of magnitude larger than any other microbial group, especially in the later stages of fermentation [>log 7.0 (CFU/g) after 24 h of fermentation]. In all fermentation variants, the final LAB counts were log 8.0–9.0 (CFU/g). The counts of the total aerobic microbiota followed the dynamics of the LAB counts closely. No filamentous fungi were found in any process. Presumptive yeasts and enterobacteria were present in low numbers in all fermentation variants [approximately log 5.0 (CFU/g)]. Yeast counts stayed relatively constant over the course of the fermentation variants, but enterobacterial counts decreased after an initial rise during the initial stages of the fermentation variants (0–36 h). This decrease in enterobacterial counts was not as pronounced in the DM2 process. AAB counts were low or below the detection limit [log 2.0 (CFU/g) for this experimental set-up] during all fermentation variants.

**FIGURE 3:**
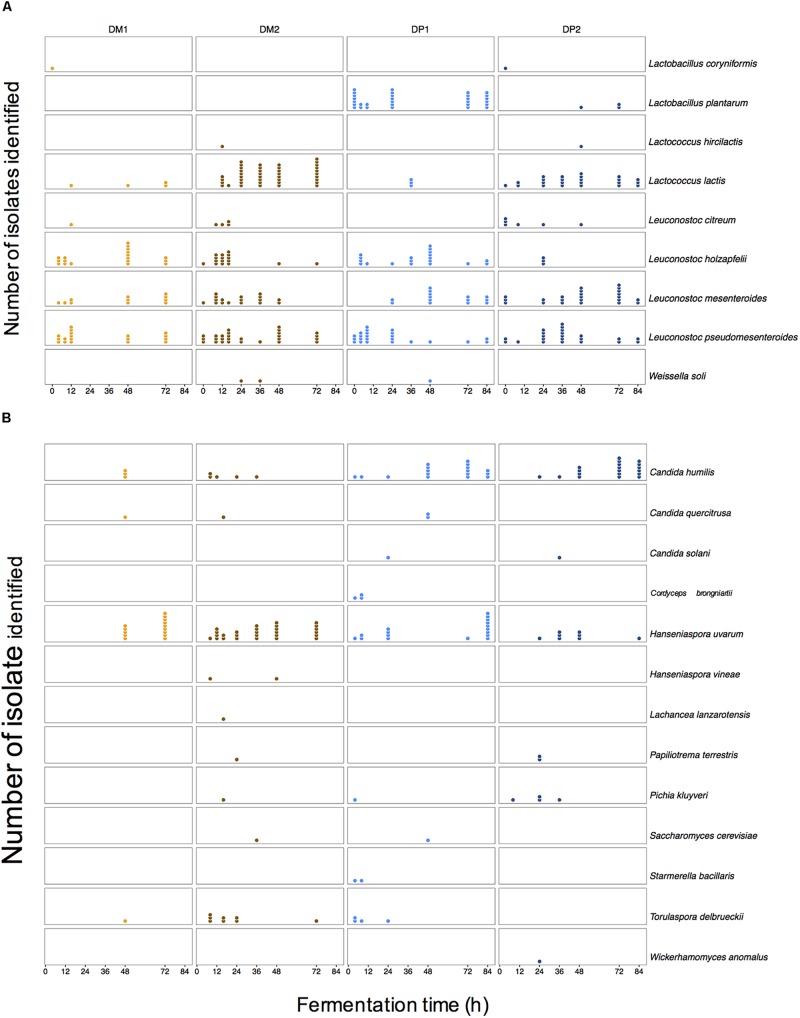
Culture-dependent microbial species diversity along the fermentation process of depulped (DP) and demucilaged (DM) beans of the Arabica coffee wet processing experiments. Colonies were identified upon isolation through plating of samples on selective agar media, followed by appropriate incubation. Lactic acid bacteria **(A)** and yeasts **(B)** were the most prevalent microbial groups in all fermentation variants of the Arabica coffee wet processing experiments. Every column represents a time series of one microbial species identified within one processing type (DM1, DM2, DP1, and DP2). Every dot represents one identification at one time point for one microbial species.

*Leuconostoc* was the most prevalent LAB genus found culture-dependently (Figure 3). *Leuconostoc pseudomesenteroides*, *Leuconostoc mesenteroides*, and *Leuconostoc holzapfelii* were the most frequent species. *Lactococcus* (especially *Lactococcus lactis*) was also frequently found during fermentation (especially in the DM processes; Supplementary Figure S2). *Lactobacillus plantarum* was highly prevalent in the DP1 process (Supplementary Figure S2). Other *Lactobacillus* species and *Weissella soli* were occasionally found during all fermentation variants. The yeasts *Candida humilis* and *Hanseniaspora uvarum* were widely found in all fermentation variants. Other yeast species, notably *Pichia kluyveri* and *Torulaspora delbrueckii*, were found only sporadically throughout all fermentation variants. The AAB *Acetobacter* and *Gluconobacter* were sparingly detected at various time points of the fermentations of the DP processes. No obvious trends in the species diversity of any microbial group were found over the course of the fermentation variants. Still, there was a tendency to find more specific species in one of the two fermentation variants. *Lactococcus lactis* (1.7 times more), *H. uvarum* (1.5 times more), and *Leuc. holzapfelii* (1.5 times more) were found more in the fermentations of the DM than in those of the DP processes. Conversely, some species were found more in the fermentations of the DP than in those of the DM processes, namely *Lb. plantarum* (not found), *C. humilis* (4.4 times more), *Leuc. mesenteroides* (1.3 times more), and *P. kluyveri* (not found). Consequently, the culture-dependent approach revealed that the microbial species diversity remained largely unchanged, whereas the size of the microbial populations evolved as fermentation proceeded.

### Culture-Independent Microbial Community Dynamics and Diversity During Fermentation

#### Metagenetics

**FIGURE 4:**
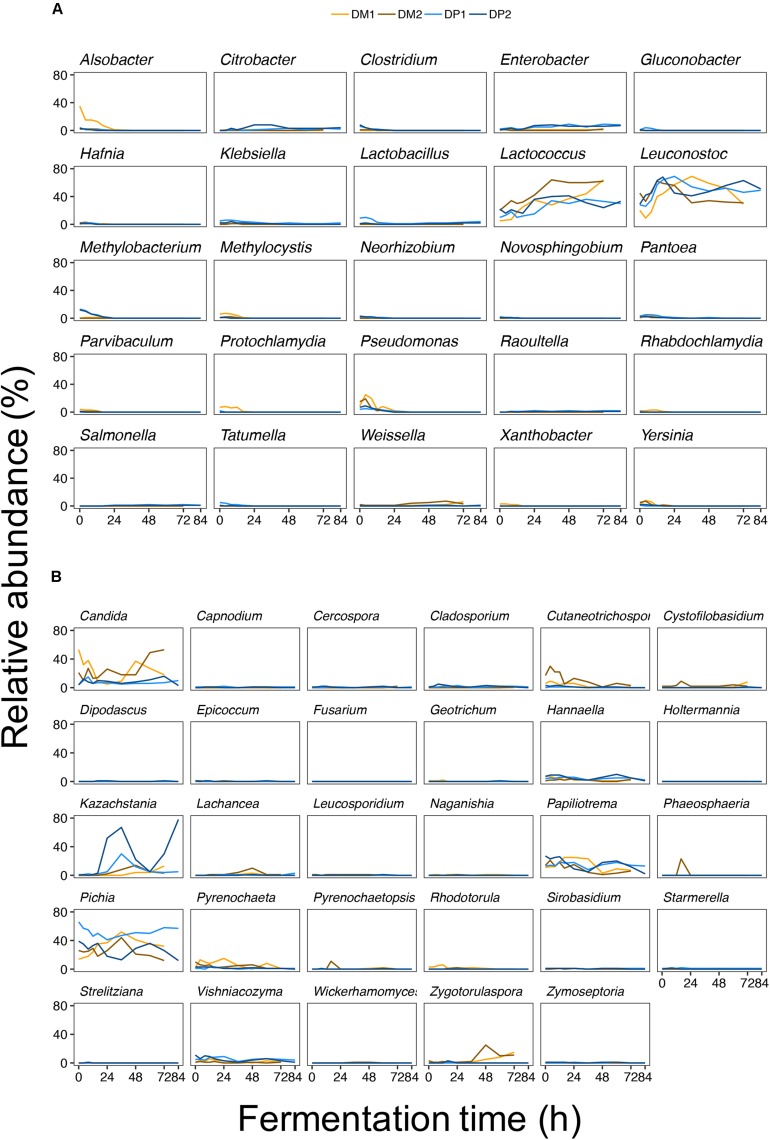
Bacterial **(A)** and fungal **(B)** amplicon sequencing variants (ASVs) detected during fermentation of depulped (DP) and demucilaged (DM) beans of the Arabica coffee wet processing experiments. To simplify visualization, individual ASVs were grouped by genus and represented in a dedicated panel. The different colors denote the different fermentation variants (blue for the DP trials and orange for the DM trials).

The major share of the bacterial sequences obtained from the fermentation variants of the DM and DP processes was allocated to LAB (Figure 4). Within this group, *Leuconostoc* and *Lactococcus* were by far the most prevalent ASVs. These ASVs had high relative abundances throughout all fermentation variants. *Lactobacillus* was only moderately present at the beginning of the fermentation of the DP1 process. *Weissella* was present solely at the end of fermentation of the DM processes. Except for the increasing relative abundance of *Lactococcus* over the course of the fermentations of the DM processes, no obvious trend was found for the LAB ASVs. Compared to the LAB ASVs, other ASVs were of minor relative abundance during the fermentation variants. These ASVs were present only in the beginning of the fermentation variants (notably *Alsobacter*, *Pseudomonas*, and *Yersinia*) or were only transiently present (such as *Citrobacter*). The fungal ASVs showed more diverse relative abundance patterns over the course of the fermentation variants. The most relative abundant ASVs over all fermentation variants were *Pichia*, *Candida*, *Kazachstania*, *Papiliotrema*, *Cutaneotrichosporon*, *Hannaella*, and *Zygotorulaspora*. Some of the fungal ASVs were more relative abundant in the fermentations of the DM processes than in those of the DP processes (in particular *Candida*, *Cutaneotrichosporon*, *Lachancea*, *Pyrenochaeta*, and *Zygotorulaspora*). In contrast, only *Hannaella* seemed to be slightly more relative abundant in the fermentations of the DP processes than in those of the DM ones. As is the case for the bacterial ASVs, no obvious trend was found for the fungal ASVs. Still, some fungal ASVs were present only in the beginning (such as *Cutaneotrichosporon*) or at the end (such as *Zygotorulaspora*) of the fermentations of the DM processes.

PERMANOVA indicated that the processing type had a significant effect on the microbial community composition. The processing type accounted for 29.93% of the variation in the culture-independent dataset (df = 1, pseudo-F = 33.83, and *p* < 10^–5^). A total of 75 from 110 ASVs were significantly discriminant between the DP and DM processes (p < 0.05). The top-ten discriminant ASVs were *Papiliotrema* 2, *Kazachstania* 1, *Lactococcus* 1, *Capnodium* 1, *Cladosporium* 2, *Citrobacter* 1, *Vishniacozyma* 1, *Zymoseptoria* 1, *Sirobasidium* 3, and *Lactobacillus* 1 (Supplementary Figure S3A). Among these ASVs, *Lactococcus* 1 was the only one that had a higher average relative abundance in the fermentations of the DM processes compared to those of the DP ones.

PERMANOVA further indicated that the fermentation duration had a significant effect on the microbial community composition. The fermentation duration accounted for 37.96% of the variation in the culture-independent dataset (df = 9, pseudo-*F* = 4.77, and *p* < 10^–5^). A total of 22 from 110 ASVs were significantly discriminant between a standard and extended fermentation duration across all fermentation variants (*p* < 0.05) (Supplementary Figure S3B). Among these ASVs, only *Lactobacillus* 2 was more prevalent after extended fermentation compared to standard fermentation duration.

#### Shotgun Metagenomics

##### Taxonomic assignment

**FIGURE 5:**
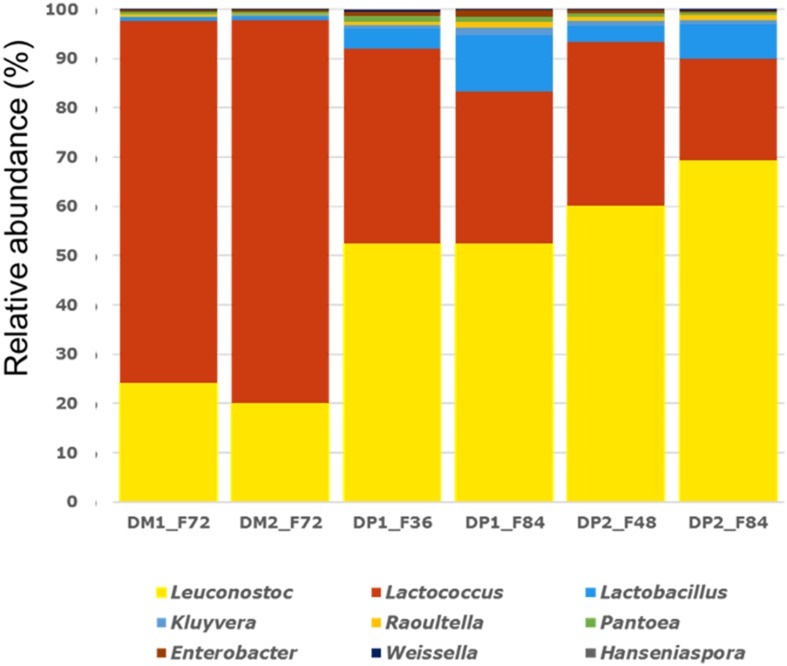
Relative abundance (%) of all microbial genera identified (>0.1%) through fragment recruitment plots on a selection of 561 bacterial and fungal genomes in the shotgun metagenomic data of the Arabica coffee wet processing experiments.

Shotgun metagenomic sequencing of the six samples selected yielded between 4,519,539 (sample DP1_F36) and 7,074,660 (sample DP2_F84) metagenomic sequences per sample, excluding *C. canephora*-related sequences. Aligning these metagenomic sequences to a customized database, representing 561 genomes, allowed taxonomic allocation of 76–87% of all reads (Figure 5).

Members of the genera *Leuconostoc* and *Lactococcus* were highly abundant in all six samples, even more pronounced in the samples obtained from the fermentations of the DM processes, for which there were relatively more metagenomic sequences related to *Lactococcus* than to *Leuconostoc*. The samples obtained from the fermentations of the DP processes had an inversed relative composition, with more sequences related to *Leuconostoc* than to *Lactococcus*, and with a substantial amount of reads related to *Lactobacillus*. The latter genus had a higher relative abundance at the end of the fermentations (DP1_F84 and DP2_F84), compared to the samples taken during the fermentations (DP1_F36 and DP2_F48), of the DP processes. All other genera with a relative abundance higher than the cut-off value of 0.1% had in fact a marginal relative abundance compared to *Leuconostoc*, *Lactococcus*, and *Lactobacillus*.

##### Functional analysis

Assembly of the metagenomic sequences using MEGAHIT, performed per sample, yielded in total 220,387 metagenomic contigs, with a length of at least 1000 bp (Table 2). Using Prokka, 459,880 protein-encoding genes were predicted and annotated, of which 46.0% (211,577) encoded known proteins. Of these proteins, 56.7% (120,003) were annotated as enzymes with an EC number, which were further used as an indication for the functional properties of the coffee fermentation ecosystem. The number of unique EC numbers per sample ranged from 880 (sample DM2_F72) till 1306 (sample DP1_F84). The number of unique EC numbers over all six samples was 1488. For each sample, a count table was composed, reflecting the number of occasions an EC number was found per sample. Next, the list of 1488 EC numbers was compacted to the sub-subclass level, resulting in a list of 176 sub-subclasses. Consequently, the counts obtained for each EC number were summed for all EC numbers within a sub-subclass per sample, representing 109,818 proteins, as the remaining number were only annotated at the subclass or class levels. The ten most occurring enzyme sub-subclasses belonged to the oxidoreductases, hydrolases, transferases, glycosidases, and ligases, and accounted for 41.7% of the 109,818 proteins (Table 3). Further, the full table with the 176 sub-subclasses (Supplementary Table S1) was used to calculate frequencies, which were used to draw a heatmap, visualizing under- and over-representation of the sub-subclasses in the six samples, scaled per sub-subclass and represented by a *Z*-score (Supplementary Figure S4). The heatmap showed a clustering based on processing type, with the two samples from the fermentations of the DM processes (DM1_F72 and DM2_F72) clustering together, but separated from the four samples of the fermentations of the DP processes. Within the latter set of samples, the two samples from the later fermentation time point of 84 h (DP1_F84 and DP2_F84) clustered together, reflecting that the different enzyme sub-subclasses were related to the type of processing.

**TABLE 2:** Overview of the number of contigs assembled with MEGAHIT, and data regarding gene prediction and annotation with Prokka for six metagenomic samples of the Arabica coffee post-harvest processing experiments.

**Sample**	**Number of contigs (>1000 bp)**	**Number of predicted genes**	**Number of hypothetical proteins**	**Number of known proteins**	**Number of known proteins with an EC number**	**Number of unique EC numbers**
DM1_F72	23,483	48,238	27,694	20,544	11,726	902
DM2_F72	23,231	50,953	29,672	21,281	12,285	880
DP1_F36	31,888	67,938	36,752	31,186	18,180	1063
DP1_F84	54,693	112,631	59,240	53,391	29,917	1306
DP2_F48	43,078	87,888	46,635	41,253	23,289	1242
DP2_F84	44,014	92,232	48,310	43,922	24,606	1189
Total	220,387	459,880	248,303	211,577	120,003	1488

*DM, demucilaging process; DP, depulping process; F, fermentation time point (in h).*

**TABLE 3:** Overview of the ten most encountered EC sub-subclasses and their functions, as obtained through functional analysis of the shotgun metagenomic data of the Arabica coffee post-harvest processing experiments.

**EC number**	**Sub-subclass function**	**Total**
1.1.1.-	Oxidoreductases, acting in the CH-OH group of donors, with NAD(+) or NADP(+) as acceptor	6074
3.6.3.-	Hydrolases, acting on acid anhydrides catalyzing transmembrane movement of substances	5783
2.7.1.-	Phosphotransferases with an alcohol group as acceptor	5735
2.1.1.-	Methyltransferases	5280
2.7.7.-	Nucleotidyltransferases	4459
2.3.1.-	Acyltransferases, transferring groups other than amino-acyl groups	4390
2.4.1.-	Hexosyltransferases	4223
3.2.1.-	Glycosidases	3599
6.1.1.-	Ligases forming aminoacyl-tRNA and related compounds	3169
3.1.3.-	Phosphoric monoester hydrolases	3108

*The total number mentioned reflects the number of proteins that were annotated with an EC number belonging to these 10 sub-subclasses for all six metagenomic samples together.*

### Dynamics of the Metabolomic Profiles of the Processing Waters

#### Fermentation Waters

**FIGURE 6:**
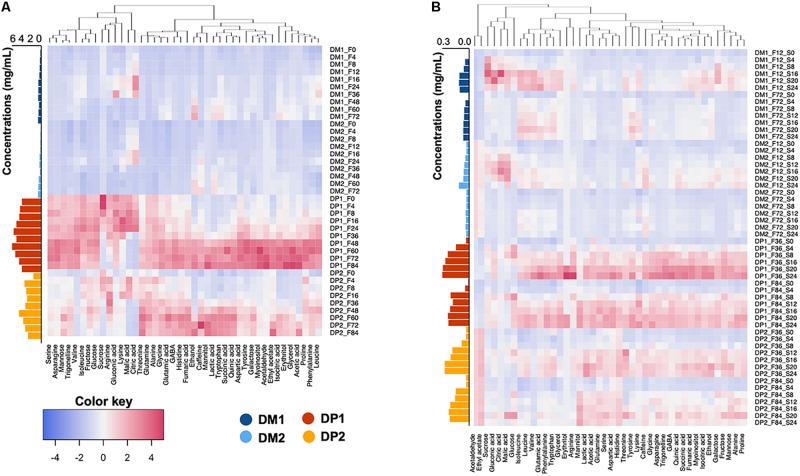
Hierarchical clustering analysis and heatmap visualization of the chemical compositional profiles of the fermentation waters **(A)** and soaking waters **(B)** from the demucilaged (DM) and depulped (DP) processes of the Arabica coffee wet processing experiments. The absolute concentrations (in mg/mL) were summed and displayed at the right side of each heatmap.

The temporal metabolomic profiles of the fermentation waters were similar among the processing types (DM and DP) and biological duplicates (first and second trials). The main differences were the absolute concentrations of the compounds targeted. The cumulative concentrations of all the compounds quantified, especially monosaccharides, organic acids, alkaloids and amino acids, were ten times higher in the fermentation waters of the DP processes than in those of the DM ones (Figure 6A). The compounds targeted could be divided into two groups based on the temporal change of their profiles during fermentation, namely rise-fall and rise-rise patterns (Figure 6A and Supplementary Figure S5A). The rise-fall pattern referred to a brief increment of their concentrations at the beginning of the fermentations, which was succeeded by a decrement toward the end. The compounds following this pattern included the simple carbohydrates sucrose, glucose and fructose, the organic acids citric acid, gluconic acid and malic acid, and the amino acids arginine, isoleucine, leucine and lysine. These compounds differed slightly in their timing to reach peak concentrations: first with sucrose (first 8 h of fermentation), followed by citric acid, malic acid, arginine, isoleucine, and lysine (after around 24 h), and then glucose, fructose, gluconic acid, and leucine (after 36 h). In addition, the concentrations of these compounds differed greatly at the end of the standard and extended fermentation durations. For example, at the end of the standard fermentations, fructose (0.5, 0.2, 6.0, and 5.4 mg/mL in DM1, DM2, DP1, and DP2, respectively) and glucose (0.3, 0.1, 4.0, and 2.6 mg/mL, respectively) had relatively high concentrations in the fermentation waters, whereas the concentrations of sucrose, citric acid, and malic acid were minimal in the fermentation waters of all four processes. Toward the end of the extended fermentations, the concentrations of fructose (0.1, 0.0, 4.6, and 2.4 mg/mL in DM1, DM2, DP1, and DP2, respectively) and glucose (0.0, 0.0, 2.0, and 1.0 mg/mL, respectively) reached lower levels. Furthermore, a small decrease of the galactose concentrations appeared at the end of the fermentations of the DM processes, which was not the case in those of the DP ones. Second, the rise-rise pattern referred to a continuous increment of the concentrations of the compounds targeted throughout the fermentation steps. This pattern covered the rest of the compounds targeted. Most of the microbial-related metabolites (e.g., acetic acid, ethanol, glycerol, mannitol, and lactic acid) started to accumulate only after 24 h or even at a later stage of fermentation. The accumulation of these compounds also occurred later in the fermentations of the DM processes than in those of the DP ones. At the end of the fermentations of the DP processes, mannitol (3.0 and 3.2 mg/mL in the first and second trials, respectively), lactic acid (1.7 and 1.8 mg/mL, respectively), and acetic acid (0.9 and 0.7 mg/mL, respectively) were the most abundant compounds in the fermentation waters. In contrast, the DM fermentation waters reached lower concentrations of mannitol (0.4 and 0.1 mg/mL, respectively), lactic acid (0.8 and 0.8 mg/mL, respectively), ethanol (0.2 and 0.1 mg/mL, respectively), and acetic acid (0.1 and 0.1 mg/mL, respectively). Plant-related compounds, such as trigonelline, caffeine, and most amino acids showed increasing concentrations along fermentation. For instance, trigonelline (0.1, 0.1, 0.3, and 0.2 mg/mL in DM1, DM2, DP1, and DP2, respectively) and asparagine (0.0, 0.0, 0.8, and 0.3 mg/mL, respectively) were among the most abundant compounds when fermentation finished. Apart from asparagine, other free amino acids, such as serine, aspartic acid, glutamine and GABA were at much higher concentrations in the fermentation waters of the DP processes than in those of the DM ones.

#### Soaking Waters

The absolute concentrations of all the compounds targeted were much lower in the soaking waters than in the fermentation waters, regardless of the processing types (DM and DP). However, the temporal metabolomic profiles of the soaking waters exhibited some differences when comparing the processes with standard and extended fermentation (Figures 6B and Supplementary Figures S5B,C). In the processes with standard fermentation duration, two dynamic patterns were found. The rise-fall pattern occurred in the case of sucrose, glucose, fructose, citric acid and malic acid, whereas the rise-rise pattern occurred for all of the other compounds targeted. In the processes with extended fermentation duration, only a rise-stable pattern was found, namely a concentration increment at the beginning of the soaking step that was followed by a relatively stable concentration. Further, differences occurred between the soaking waters of the DM and DP processes. The cumulative concentrations in the soaking waters were slightly higher for the DP processes than in those for the DM ones, and were contributed by the presence of microbial metabolites (lactic acid, mannitol, and succinic acid), alkaloids (trigonelline and caffeine), and some amino acids (aspartic acid, arginine, serine, and GABA). In the DP soaking waters, the most abundant compounds at the end of the soaking step were mannitol, lactic acid, fructose, and ethanol in the processes with both standard and extended fermentation durations (Supplementary Figures S5B,C). The mannitol concentrations at the end of the soaking step were lower in the processes with standard fermentation duration (0.10 and 0.13 mg/mL for DP1 and DP2, respectively) than in the processes with extended fermentation duration (0.29 and 0.27 mg/mL, respectively). In contrast, the concentrations of lactic acid (0.19–0.34 mg/mL for the DP processes), fructose (0.13–0.38 mg/mL), and ethanol (0.03–0.09 mg/mL) were higher in the processes with standard fermentation duration than in those with extended fermentation duration. In the DM soaking waters, the most prevalent compounds at the end of the processing were lactic acid (0.04–0.14 mg/mL for the DM processes), ethanol (0.008–0.041 mg/mL), acetic acid (0.007–0.023 mg/mL), and mannitol (0.002–0.017 mg/mL).

### Dynamics of the Metabolomic Profiles of the Coffee Beans

#### Fermenting Beans

**FIGURE 7:**
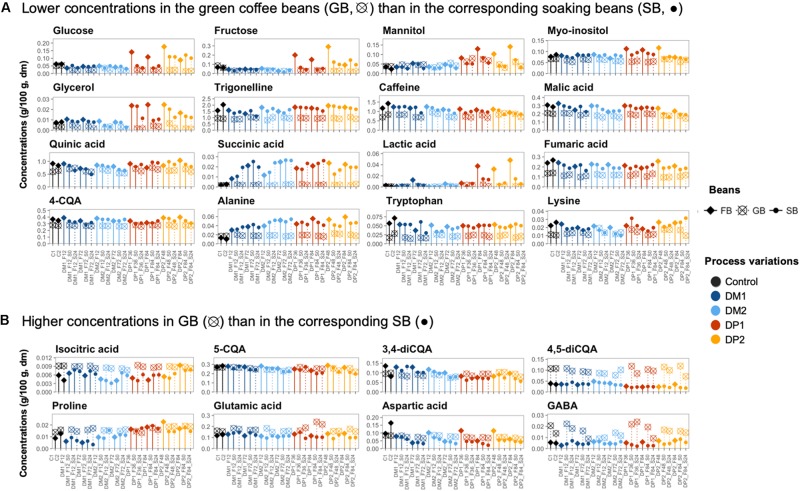
Comparison of the chemical compounds in the coffee bean samples of different processing steps of the Arabica coffee wet processing experiments. The selected compounds were present at either lower concentrations in the green coffee beans (GB) than in the corresponding soaking beans (SB) **(A)** or higher concentrations in the GB than in the corresponding SB **(B)**. The selected bean samples represent the end of the fermentations (FB; ◆), before and after soaking (•), and their corresponding GB (⊗) across the different processing variants. The freshly demucilaged beans and the GB produced in the control processes (C, black) are also included. Only the chemical compounds with consistent trends are shown.

The temporal metabolomic profiles of the fermenting beans from the DM and DP processes were similar. The compounds targeted could be divided into four groups, based on the temporal change of their profiles, namely *(i)* an off-phase evolution (in the case of sucrose versus the monosaccharides glucose and fructose), *(ii)* a rising trend, *(iii)* a decreasing trend, and *(iv)* a relatively stable concentration along the fermentations (Figure 7 and Supplementary Figure S6A).

Concerning the first group, the glucose concentrations in the fermenting beans always evolved in the same phase as those of fructose, but off-phase with those of sucrose. This took place in the DM and DP processes. However, the glucose (0.062, 0.068, 0.220, and 0.200 g/100 g for DM1, DM2, DP1 and DP2, respectively) and fructose concentrations (0.062, 0.063, 0.360, and 0.310 g/100 g, respectively) were more than three times higher in the DP beans than in the DM ones at the start of the fermentations (Supplementary Figure S6A). Yet, such differences were reduced toward the end of the extended fermentation durations, as the glucose and fructose concentrations decreased in the DP beans along the fermentations. Concerning the second group (rising trend), the concentrations of mannitol, succinic acid, lactic acid, alanine, and glycine were positively correlated with the fermentation time (*p* < 0.05). Among these compounds, the concentration build-ups of mannitol (0.13 and 0.14 g/100 g after 84 h of fermentation for DP1 and DP2, respectively) and lactic acid (0.038 and 0.048 g/100 g, respectively) were higher with the DP processes, resulting in averages that were three and six times higher than those in the DM beans, respectively, when fermentation finished. The rest of the compounds showed comparable concentrations for the DM and DP processes. For example, the succinic acid (0.021, 0.025, 0.021, and 0.022 g/100 g for DM1, DM2, DP1, and DP2, respectively) and alanine concentrations (0.038, 0.052, 0.056, and 0.059 g/100 g, respectively) were similar in the DM and DP beans at the end of the extended fermentation durations. Concerning the third group (decreasing trend), the concentrations of malic acid, citric acid, gluconic acid, caffeine, aspartic acid, arginine, and histidine were negatively correlated with the fermentation time (*p* < 0.05). Among these compounds, the decrease of the caffeine and citric acid concentrations was more associated with the DM processes. The concentrations of arginine (0.012, 0.010, 0.022, and 0.024 g/100 g for DM1, DM2, DP1, and DP2, respectively, at the end of the extended fermentation durations) were higher in the DP beans than those in the DM ones, whereas all the other compounds showed comparable concentrations for the two processing types. Concerning the fourth group (stable concentrations), the concentrations of some amino acids (e.g., proline, isoleucine, and tyrosine) were higher in the DP beans than in the DM ones, whereas the concentrations of the three diCQA isomers were higher in the DM beans than in the DP ones.

#### Soaking Beans

After the washing step, the concentrations of certain compounds decreased in the coffee beans (Figure 7 and Supplementary Figures S6B,C). For example, the concentrations of glucose, fructose, mannitol, and lactic acid decreased, especially in the DP processes. The lactic acid and mannitol concentrations were even reduced, down to 1/3 and 1/20 after washing, respectively.

Along the 24-h soaking step, the metabolomic profiles of the soaking beans remained relatively stable, despite that certain compounds experienced concentration changes depending on the processing type (DM and DP) and the fermentation duration (standard and extended; Supplementary Figures S6B,C). In the processes with standard fermentation duration, the succinic acid concentrations were positively correlated with the soaking time in both the DM and DP processes, whereas the aspartic acid and arginine concentrations were negatively correlated (*p* < 0.05). In the processes with extended fermentation duration, lactic acid and mannitol had higher initial concentrations compared to the processes with standard fermentation duration, but their concentrations diverged to similar levels toward the end of soaking in both the DM and DP processes. When comparing the bean compositions between the start and the end of soaking, the concentrations of mannitol, glycerol, citric acid, malic acid, quinic acid, lactic acid, asparagine, and aspartic acid were higher at the start of soaking, whereas the concentrations of succinic acid and glycine were higher at the end of soaking.

A PCA based on a correlation matrix included the metabolomic data of the FB and SB from the DM and DP processes (Supplementary Figure S7A). Two PCs were obtained, explaining 41% of the total variance. PC1 was characterized by positive loadings of certain amino acids (isoleucine, tyrosine, proline, leucine, phenylalanine, etc.), glycerol, glucose, fructose, mannitol, acetic acid, and lactic acid, as well as negative loadings of 4,5-diCQA, 3-CQA, 3,4-diCQA, caffeine, and citric acid. PC2 was characterized by positive loadings of succinic acid, acetic acid, alanine, and isocitric acid, as well as negative loadings of aspartic acid, serine, fumaric acid, asparagine, malic acid, sucrose, and fructose. Based on these two PCs, the DP beans were more associated with the positive values of PC1, and the DM beans were more associated with the negative values of PC1. The FB were more associated with the negative values of PC2, and the SB were more associated with the positive values of PC2.

### Green Coffee Beans Produced by Different Processing Variants

#### Quantitative Analyses

Compared to the start of drying, most metabolites tended to degrade during drying, with several exceptions (Figure 7 and Supplementary Figure S8). The corresponding GB contained lower concentrations of glycerol, fumaric acid, lactic acid, succinic acid, trigonelline, alanine, and tryptophan (Figure 7A), as well as higher concentrations of compounds such as isocitric acid, 4,5-diCQA, aspartic acid, GABA, glutamic acid, proline, and serine (*p* < 0.05) (Figure 7B). In the control processes, the concentrations of glucose, fructose, succinic acid, mannitol, lactic acid, and alanine did not show significant differences before and after the drying step.

The GB produced from the control processes contained higher concentrations of glucose, fructose, citric acid, malic acid, asparagine, and aspartic acid than all the other GB processed from the DM and DP processes (Figure 7). In contrast, the concentrations of mannitol, succinic acid, lactic acid, alanine, tyrosine, proline, glutamic acid, and glutamine were higher in the DM and DP beans than in the control GB, whereas beans from the DP1 process had the highest concentrations of mannitol and lactic acid. Some compounds, including citric acid, malic acid, gluconic acid, isocitric acid, alanine, isoleucine and aspartic acid, had decreasing concentrations with long underwater times. Other compounds, including quinic acid, caffeine, 4-CQA, 5-CQA, and glutamic acid had decreasing concentrations with long underwater times in the DM processes but not in the DP ones.

**FIGURE 8:**
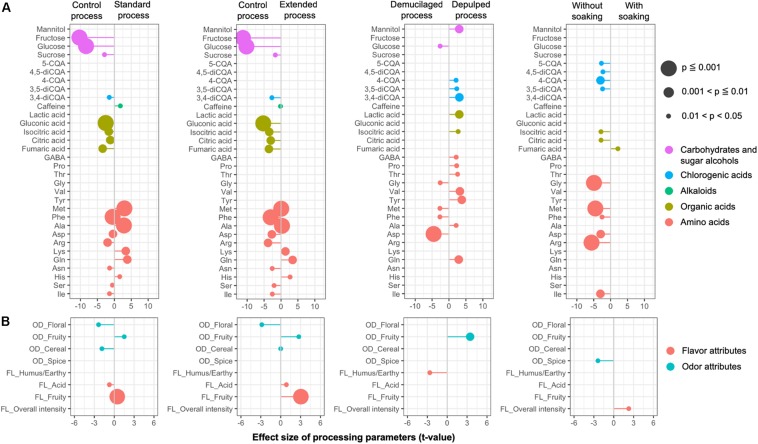
Impacts of the processing parameters of the Arabica coffee wet processing experiments on the chemical compositions of the green coffee beans **(A)** and the sensory profiles of the brewed coffees **(B)**, based on the effective size (*t*-value) generated from linear mixed effect models. Only the chemical compounds and sensory attributes with a probability level less than 0.05 are listed. The chemical compounds from different chemical groups **(A)**, as well as the flavor and odor attributes from the sensory analyses **(B)**, are indicated in different colors. OD, odor; FL, flavor.

LMEM were established to test the effect of different processing variants on the metabolite concentrations in the GB, namely the processing type, fermentation duration, and soaking (Figure 8A). The linear model indicated a strong impact of the fermentation duration (21 compounds with *p* < 0.05), followed by the processing type (18 compounds with *p* < 0.05), and a soaking step (13 compounds with *p* < 0.05). A long fermentation duration had a significant negative impact on the concentrations of fructose, glucose, gluconic acid, isocitric acid, citric acid, fumaric acid, succinic acid, 3,4-diCQA, and certain amino acids (including methionine, phenylalanine, alanine, aspartic acid, arginine, asparagine, serine, and isoleucine), and positive impacts on the concentrations of glutamine, histidine, and lysine. The DP processes, compared to the DM ones, had significant negative impacts on the concentrations of glucose and certain amino acids (including aspartic acid, methionine, phenylalanine, and glycine), but positive impacts on the concentrations of lactic acid, mannitol, isocitric acid, 3,4-diCQA, 3,5-diCQA, 4-CQA, and certain amino acids (including tyrosine, valine, glutamine, threonine, proline, alanine, and GABA). The application of an extra soaking step had significant negative impacts on the concentrations of isocitric acid, citric acid, 4-CQA, 5-CQA, 3,5-diCQA, 4,5-diCQA, and certain amino acids (including arginine, glycine, methionine, isoleucine, aspartic acid, and phenylalanine), and a significant positive impact on the fumaric acid concentration.

A PCA based on a correlation matrix of the same dataset resulted in two PCs, explaining 42% of the total variance (Supplementary Figure S7B). PC1 was characterized by positive loadings of mannitol, lactic acid, quinic acid, glutamic acid, isoleucine, and proline, and negative loadings of galactose, myo-inositol, serine, aspartic acid, and asparagine. PC2 was characterized by negative loadings of gluconic acid, arginine, 3,4-diCQA, and 4,5-diCQA. The GB from the control processes were positioned at the negative values of PC1 and PC2. The GB produced from the DP1 process were more associated with the negative values of PC1 and the positive values of PC2. The extended-processed GB of both the DM and DP processes were more associated with the positive values of PC2, whereas the standard-processed ones were more associated with the negative values of PC2.

#### Semi-Quantitative Volatile Profiling

**FIGURE 9:**
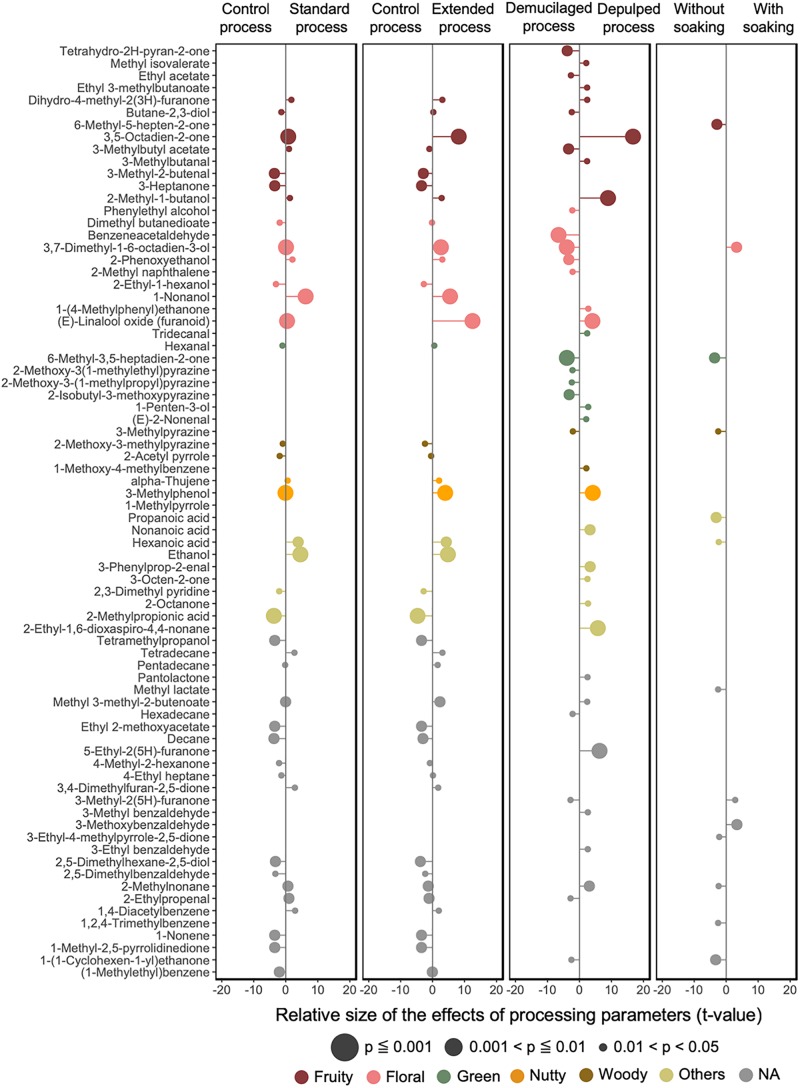
Impacts of the processing parameters of the Arabica coffee wet processing experiments on the volatile profiles of the green coffee beans, based on the effective size (*t*-value) generated from linear mixed effect models. Only the volatiles with a significance value less than 0.05 are listed. The odor type of each of the volatiles is indicated with a different color.

Around 200 compounds were found in the GB samples. The most abundant groups were aliphatic alcohols, aliphatic alkanes, and benzene derivatives, followed by aliphatic aldehydes, heterocyclic compounds, and aliphatic ketones. Terpenes, sulfur-containing compounds, and lactones were present in lower abundances. The total aroma intensities of the standard-processed GB of the DM processes were lower compared to the ones from other processing variants. The application of soaking decreased the total aroma intensities, except for the extended-processed GB from the DP2 process (Figure 9).

LMEM were established to test the effect of different processing variants on the compounds targeted in the volatile profiles of the GB, namely the processing type, fermentation duration, and soaking (Figure 9). The linear model indicated a strong impact of the processing type (43 volatile compounds with *p* < 0.05) and fermentation duration (40 volatile compounds with p < 0.05), followed by the application of a soaking step (13 compounds with *p* < 0.05). The DP processes had significant positive impacts on volatile compounds such as 3,5-octadien-2-one (fruity), 2-methyl-1-butanol (fruity), 5-ethyl-2(5H)-furanone, 3-methylphenol (woody), and trans-linalool oxide (floral) (with *p* < 0.001), and negative impacts on volatile compounds such as benzeneacetaldehyde (floral), 3,7-dimethyl-1,6-octadien-3-ol (linalool, floral), and 6-methyl-3,5-heptadiene-2-one (green) (with *p* < 0.001). The longer fermentation durations had significant positive impacts on trans-linalool oxide (floral), 3,5-octadien-2-one (fruity), 1-nonanol (floral), ethanol (alcohol), 3-methylphenol (woody), and linalool (floral), and negative impacts on 1-methyl-1H-pyrrole (woody) and 2-methylpropanoic acid (buttery) (*p* < 0.001). The application of soaking gave positive impacts on linalool (floral) and negative impacts on 6-methyl-3,5-heptadien-2-one (green), 6-methyl-5-hepten-2-one (fruity), and propanoic acid (cheesy).

### Sensory Analysis

The scores for the sensory notes were close to each other across the samples. LMEM were established to evaluate the effect of different processing variants on the sensory profile, namely the processing type, fermentation duration, and soaking (Figure 8B). The linear model indicated a strong impact of the fermentation duration (five parameters with *p* < 0.05), followed by the processing type (two parameters with *p* < 0.05), and a soaking step (two parameters with *p* < 0.05). A long fermentation duration had a positive impact on the fruity (OD and FL) and acidity (FL) notes and a negative impact on the cereal (OD) and floral (OD) notes. Compared to the DM processes, the DP processes had a positive impact on the fruity (OD) notes and a negative impact on the humus/earthy (FL) notes. The application of soaking had a positive impact on the overall flavor intensity and a negative impact on the spicy (OD) notes.

According to the PCA analysis, two PCs covered 48% of the data variability (Supplementary Figure S7C). Smoothness (TX) was more associated with the positive values of PC1, whereas bitter and roasty (FL) notes were more associated with the negative values of PC1. Fruity and acidic (FL) notes, as well as fruity, burnt, and cereal (OD) notes were more associated with the negative values of PC2. The samples from the processes with extended fermentation duration were associated with the negative values of PC1, whereas the samples from the processes with extended fermentation duration and the control processes were associated with the positive values of PC1. No major separations were found between the processing types (DM and DP).

### Metabolite Diffusion From Different Coffee Substrates

**FIGURE 10:**
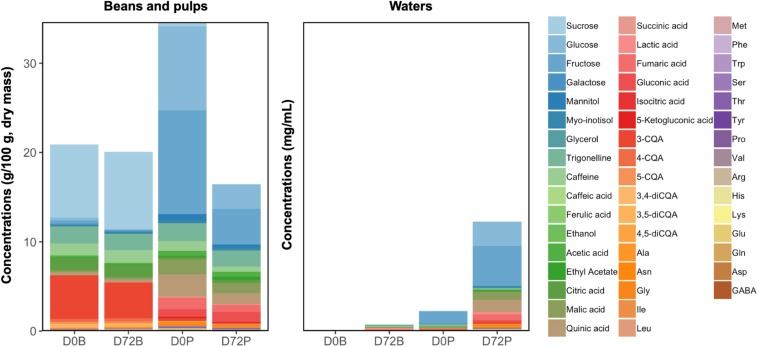
Compositional changes in the demucilaged beans and pulps, and in their corresponding water samples, before and after 72 h of soaking in separate buckets. D0B and D72B refer to the water and bean samples from the diffusion tests of the demucilaged beans at 0 and 72 h, respectively, whereas D0P and D72P refer to the water and pulp samples from the diffusion tests of the pulps at 0 and 72 h, respectively.

The beans and pulps from the fresh coffee cherries displayed different metabolomic profiles, whereby the pulps contained more glucose, fructose, mannitol, some organic acids (malic acid, quinic acid, succinic acid, isocitric acid, gluconic acid, and 5-ketogluconic acid), and certain amino acids (arginine, asparagine, serine, glutamic acid, and glutamine). The soluble portions of both beans and pulps decreased after 72 h of submersion, whereby the decrease was more extensive in the pulps (Figure 10). The concentrations of glucose, fructose, trigonelline, malic acid, 3-CQA, 3,5-diCQA, 4,5-diCQA, aspartic acid, and asparagine were lower in the beans after submersion, whereas the concentrations of ethanol, mannitol, lactic acid, succinic acid, alanine, glutamic acid, and GABA increased in the beans after submersion. In the pulps, most of the compounds targeted had lower concentrations after submersion, except for ethyl acetate, lactic acid, 4-CQA, 5-CQA, asparagine, glycine, and GABA.

Furthermore, the loss of the soluble portions of the beans and pulps corresponded to the increments of metabolites in their surrounding waters. In the case of the pulp waters, glucose, fructose, myo-inositol, malic acid, quinic acid, fumaric acid, alanine, asparagine, aspartic acid, glutamic acid, GABA, and serine reached higher concentrations compared to those in the bean waters. Compounds such as ethanol and lactic acid reached similar concentrations after 72 h in both the bean and pulp waters.

## Discussion

In the past, the fermentation step of wet coffee processing has been studied, mainly from a microbiological point of view, by means of both cultivation-based methods and culture-independent approaches (Silva et al., 2008; Vaughan et al., 2015; Feng et al., 2016; Hamdouche et al., 2016; De Bruyn et al., 2017; Pereira et al., 2017; Waters et al., 2017; Zhang et al., 2019). Indeed, most of these studies focused on the species diversity and in some cases the activities of the coffee bean microbiota to try to explain green coffee bean production, yield, and quality. The present study tackled the dynamics of the microbial communities and metabolomic profiles of the coffee beans, processing waters (fermentation and soaking waters) and green coffee beans, and the sensory quality of the coffee brews, thereby evaluating the impacts of multiple processing parameters (demucilaging and depulping, fermentation duration, and soaking).

Both demucilaged and depulped processing methods are commonly applied by coffee producers, depending on the water resources and infrastructure available (Murthy and Naidu, 2012). The main technical difference between these two processes, i.e., the amount of mucilage attached to the beans at the start of the fermentation step, was translated into differences in the microbial community compositions and in the metabolite concentrations present in the fermentation waters. As a result, the DP processes contained a richer nutrient reservoir for the microorganisms and plant enzymes present, resulting in stronger fermentation effects for the DP processes than for the DM ones. Nevertheless, the comparable temporal profiles of the metabolites in the fermentation waters of both processing types implied similar microbial activities, plant material diffusion dynamics, and enzymatic reactions. The mucilage of matured coffee cherries is copious in carbohydrates, organic acids, alkaloids, and free amino acids (Neu et al., 2016; Zhang et al., 2019). As the main plant material present in the fermentation waters, these compounds gradually diffused into the surrounding water, due to steep concentration gradients (Cheng et al., 2016). Along with the increasing concentrations of nutrients in the fermentation waters, some chemical compounds were used by the microorganisms as substrates to be fermented or converted into diverse microbial metabolites.

LAB, in particular mesophilic species, were predominant from the beginning of the fermentations, albeit in lower counts than what was found in previous similar experiments carried out in Ecuador (De Bruyn et al., 2017; Zhang et al., 2019). In contrast to these previous experiments, AAB appeared sporadically, whereas yeasts showed no major changes in counts. This low prevalence of microorganisms might be due to environmental factors, such as the temperature, which was rather low compared to other coffee fermentation experiments performed before (De Bruyn et al., 2017; Zhang et al., 2019), or due to intrinsic factors, such as the coffee variety used (Brando and Brando, 2014; Silva, 2014; Waters et al., 2017; Pereira et al., 2019). Furthermore, the microbial community compositions were affected by both the processing type and fermentation duration. Among the LAB, species of *Leuconostoc* seemed to be prevalent during all fermentations, whereas other communities appeared to be more associated with fermentations of either the DM or DP processes. These tendencies were confirmed through amplicon sequencing, elucidating that microbial communities were distributed differently, although largely shared between fermentations of the DM and DP processes. Based on shotgun metagenomic sequencing, a more detailed insight was obtained, as *Leuconostoc* had a much higher relative abundance in the fermentations of the DP processes compared to those of the DM ones. In the latter fermentations, *Lactococcus* was present in higher relative abundances.

Microbial communities preferentially associated with one processing type could be more adapted to the concomitant conditions (i.e., substrate concentrations), which inferred a competitive advantage for these communities and thus increased the relative abundances of their respective ASVs. Corresponding to the metabolite composition differences in the DM and DP fermentation waters, genera performing well during the fermentations of the DM processes could be less fastidious or more efficient in nutrient uptake, because part of the mucilage was stripped away mechanically during demucilaging, which translated into faster-growing LAB genera that outcompeted slower-growing ones. To that effect, a specific *Lactococcus* ASV seemed to be well-adapted to the lower substrate concentrations in the fermentation waters of the DM processes. Conversely, a specific *Lactobacillus* ASV was present only in the comparatively high-nutrient conditions of the fermentation waters of the DP processes. This was partly reflected in the isolate identifications as well, as specific DM samples had particularly high factor loadings in the PCA for *Lc. lactis* and, conversely, specific DP samples had particularly high factor loadings in the PCA for *Lb. plantarum.* The fermentation duration also affected the microbial community compositions significantly. Notably, a specific *Lactobacillus* ASV increased in relative abundance when the fermentation duration was extended. Other ASVs that were significantly discriminant between standard and extended fermentation durations decreased as the fermentation duration increased. As mentioned above and compared to other coffee fermentations in which LAB occurred in relatively high counts, the present study encountered a higher prevalence of *Lactococcus* and a lower prevalence of *Lactobacillus* (De Bruyn et al., 2017; Zhang et al., 2019). This could be due to the relative low environmental temperature during the current experiments. Indeed, lactococci can grow at temperatures as low as 10°C, whereas lactobacilli have a broad temperature range in which they can grow, their optimal growth temperatures ranging from 30–40°C (Kim, 2014; Pot et al., 2014).

All the LAB species identified fermented the mucilage carbohydrates to lactic acid, ethanol, acetic acid, and/or mannitol through the Embden-Meyerhof-Parnas pathway (homofermentative LAB) or phosphoketolase pathway (heterofermentative LAB) (Zaunmüller et al., 2006; Gänzle, 2015). Malic acid could be converted into lactic acid through malolactic fermentation, whereas citric acid could be converted into lactic acid and acetic acid, following pyruvate metabolism, or succinic acid through the reductive branch of the tricarboxylic acid cycle (Gänzle, 2015). Branched-chain amino acids (isoleucine and leucine), arginine, and lysine could be converted into the corresponding α-keto acids through transamination, or further to the corresponding aldehydes and alcohols through consecutive decarboxylation and dehydrogenation (Fernández and Zúñiga, 2006; Gänzle, 2015). Finally, due to prolonged fermentation, a successive pattern of substrate consumption occurred, i.e., first sucrose, followed by monosaccharides, organic acids, and branched-chain amino acids. Consequently, the higher substrate concentrations in the fermentation waters of the DP processes yielded a higher accumulation of microbial metabolites (especially lactic acid and mannitol) in these fermentation waters compared to those of the DM ones, which gave a greater fermentation effect on the fermenting beans.

Further, the metabolite compositions of the fermenting beans were modified through the combination of microbial activities and the endogenous bean metabolism. Indeed, multiple microbial metabolites accumulated onto the fermenting beans upon fermentation, especially lactic acid and mannitol. This was more pronounced in the fermentations of the DP processes, demonstrating the positive effects of microbial activities during coffee fermentation, especially with abundantly present substrates and long fermentation durations. Further, many compounds in the beans were affected by their endogenous metabolism, due to hypoxia. Under hypoxia, the anaerobic ethanol and lactate fermentations and the GABA shunt are activated in the beans, due to oxygen-limiting conditions (Bytof et al., 2005). Aspartic acid can be converted into oxaloacetate via aspartate aminotransferase, and glutamic acid into α-ketoglutarate via glutamate dehydrogenase or GABA via glutamate decarboxylase (Fait et al., 2007; Hildebrandt et al., 2015). Stimulated by prolonged oxygen deprivation, GABA can be further converted into succinic acid, to participate in the tricarboxylic acid cycle, simultaneously producing alanine from pyruvate (Haäusler et al., 2014; Snowden et al., 2015). This corroborated with decreasing aspartic acid and increasing succinic acid and alanine concentrations in the beans along the fermentations, as reported before in other dicotyledons (Limami et al., 2008; Sato et al., 2016). In addition, the off-phase pattern of sucrose and its constituting monosaccharides glucose and fructose confirmed the diverse equilibrium and remobilization of the carbohydrate resources in the beans (De Bruyn et al., 2017; Zhang et al., 2019). Although present at lower abundances than LAB, the contribution of yeasts to changes in the fermentation waters, for instance, consumption of carbohydrates and branched-chain amino acids as well as fusel alcohol and ester production through Ehrlich pathway, cannot be excluded.

After the fermentation step, the washing and soaking steps removed the liquefied mucilage from the beans and also facilitated drying (Brando and Brando, 2014). However, compounds of microbial origin diffused from the fermenting beans into the clean surrounding waters and this was more pronounced in the DP processes and/or processes with extended fermentation duration. The distinct speeds of metabolite accumulations between the processes with standard and extended fermentation durations were the consequences of the concentration gradients present. Larger concentration gradients in the processes with extended fermentation duration accelerated the diffusion of metabolites into the soaking waters, whereas processes with standard fermentation duration showed a more gradual accumulation. In contrast, the starting concentrations of the microbial substrates (especially sucrose, glucose, citric acid, and malic acid) were higher in the soaking waters of the processes with standard fermentation duration, allowing the microorganisms to metabolize them, and resulting in a concomitant increment of the lactic acid concentrations. The fermentation effects on the coffee beans were lessened after washing and soaking. Only a small fraction of the microbial metabolites (in the DM and DP processes) or glucose and fructose (in the DP processes) were retained on the beans after soaking. Consequently, the concentration differences of the microbial metabolites between the different processing variants were more obvious on the coffee beans without soaking than on the ones after soaking. Furthermore, an active endogenous bean metabolism during soaking continued to modify the bean compositions, especially concerning the concentrations of alanine, aspartic acid, and succinic acid.

The diffusion tests of the coffee substrates suggested that both the beans and pulps could lose their soluble portions into the surrounding water after prolonged underwater submersion. Compared to the beans, the soluble portions diffused more easily from the pulps, probably due to the more porous and fleshy structure of the pulps (Borém et al., 2014). This might explain some local practices that add pulp to the fermentation tank to create greater fermentation effects, as the pulp can be used as an additional substrate by the microorganisms. In the case of pulp-free demucilaged beans, the loss of the soluble portions of the beans and the accumulation of metabolites in the surrounding water implied that a certain degree of diffusion also took place during prolonged underwater submersion. In combination with the endogenous bean metabolism and microbial activities, all of these factors could modify the metabolite compositions of the beans during water submersion.

The drying stage is the stage generating the final green coffee beans, during which the beans gradually lose their metabolic activities, with a concomitant moisture drop and microbial activities of less significance (Waters et al., 2017; Zhang et al., 2019). By comparing coffee beans before and after drying, differences in their metabolite compositions occurred, due to both chemical degradation and seed responses to drought stress (Bytof et al., 2005; Rendón et al., 2013). In response to the dehydration stress, amino acids such as GABA, proline, aspartic acid, and glutamic acid accumulated in the drying beans as a result of an increased biosynthesis to maintain cell membrane stability (Bytof et al., 2005; Farooq et al., 2009). The decreasing concentrations of fumarate, malate, and succinate and the increasing concentrations of isocitrate implied responses of tricarboxylic acid cycle intermediates under dehydration and seed respiration (Selmar et al., 2006; Bai et al., 2012). The reduction of monosaccharides, amino acids, and other organic acids could also be caused by a persistent metabolism in the beans during prolonged drying. Furthermore, interconversions and degradation of CGAs were reflected in changes of the quinic acid and CQA concentrations and ratios between the CGA isomers in the beans. This also corresponded to a loss of total CQAs that occurred during drying, confirming previous results (De Bruyn et al., 2017; Zhang et al., 2019). Such continuous changes in the bean compositions underlined the importance of the drying practices and durations on-farm, where either good or poor practices would have an impact on the final green coffee bean quality.

As many changes took place along the processing, the metabolite compositions of the green coffee beans were inevitably affected by the processing parameters, primarily the fermentation duration and processing type. The green coffee beans from the control processes, which were actually equivalent to semi-dry processing, retained higher levels of volatiles and non-volatiles compared to the wet-processed ones. The remnants of the mucilage and absence of underwater submersion effectively reduced the potential loss of substances and dry materials, caused by diffusion and abiotic stress (Borém et al., 2014). In contrast, the wet-processed green coffee beans were subjected to the effects of both underwater fermentation and endogenous bean metabolism. As addressed during previous studies, the influence of the fermentation duration on the green coffee beans was associated with the presence of substrates from the mucilage and microbial activities (De Bruyn et al., 2017; Zhang et al., 2019). A high mucilage amount, as seen in the fermentation waters of the DP processes, reinforced the fermentation effect and could be retained on the green coffee beans, especially in the absence of soaking. The application of soaking facilitated the removal of the fermentation effects, as reflected in a significant loss of both volatile and non-volatile compounds. Furthermore, these processing parameters also affected the volatile profiles of the green coffee beans and could be linked to the cup quality (Gonzalez-Rios et al., 2007). The volatiles related to floral and fruity notes of the green coffee beans seemed to be enhanced by a long fermentation duration. The volatiles related to floral notes also appeared at higher relative abundances in the DM-processed green coffee beans, whereas the fruity-related volatiles appeared more in the DP-processed ones. Most of these compounds might survive the roasting process and, hence, modify the sensory profiles of the brewed coffees. Moreover, the loss of these substances tended to be more obvious for the DM-processed green coffee beans than for the DP-processed ones. This could be credited to a lack of extra barriers on the beans and a larger concentration gradient in the fermentation step of the DM processes compared to the DP ones. Also, the reproducibility of the experiments was better with the DM processes than with the DP ones regarding both the processing water and bean compositions, implying that the former processing type was less dependent on the ambient temperature, water to bean ratio, or other technical aspects. Lastly, the extra soaking step mainly functioned as a cleaning system, whereby both volatile and non-volatile compounds were lost into the surrounding water, generating cleaner green coffee beans. This might explain the empirical findings that soaking of fermented washed beans could deliver a cleaner note to the cup compared to non-soaked ones (Velmourougane, 2011; Murthy and Naidu, 2012).

The sensory profiles of the final coffees were the combined result of roasting and brewing. Roasting changes the appearance, physical structure, and chemical composition of green coffee beans, while brewing extracts soluble compounds from the flavorful roasted coffee beans into water (Poisson et al., 2017). The low-molecular-mass compounds in the green coffee beans, such as simple carbohydrates, free amino acids, trigonelline and CGAs, are the key precursors during roasting and, hence, undergo chemical reactions, whereas the high-molecular-mass compounds are hardly affected by roasting but contribute to the texture and mouthfeel of the brewed coffee (de Rosa et al., 2016; Pereira et al., 2019). Although the aroma variation of the brewed samples was constrained, certain attributes still exhibited differences among the processing variants. Similar to the case of the green coffee beans, the fermentation duration had the greatest impact on the sensory quality of the brews, followed by the processing type and the application of a soaking step. The higher acidity and fruity notes of the brewed coffees, which were generally desired, were enhanced by prolongation of the fermentation step and the application of a depulping process. However, the floral notes were more prominent in the control processes, which could be related to the higher concentrations of amino acids and organic acids present in the green coffee beans. The application of a more ecological demucilaging process did not result in striking differences in the cup quality, compared to depulped processing, which was in accordance with the findings of previous studies carried out at different locations (Brando and Brando, 2014). However, there is still potential for a depulped process in combination with soaking to lower the earthy and spicy notes, which are normally related to Robusta beans or low-quality Arabica beans (Borém et al., 2014). In the present study, the Catimor variety used, a hybrid of the Caturra and Timor varieties, inevitably carried a certain degree of flavor inheritance from its Robusta ancestor (Sakiyama and Ferrão, 2014). Therefore, the use of depulping and soaking could provide a margin for quality improvement of such varieties from a processing perspective.

Lastly, the effect of terroir, the coffee variety available, and the geographical location should also be considered when evaluating the degree of influence of the processing parameters on the coffee quality. Compared to a previous study with *C. arabica* var. Typica on an Ecuadorian plantation (Zhang et al., 2019), the initial microbial load, the microbial community dynamics of the fermentation step, and the metabolite compositions of the coffee beans and processing waters differed. The microbial load of the coffee cherries was lower with *C. arabica* var. Catimor in the present study, compared to the Typica variety used in a previous study (Zhang et al., 2019). In the latter case, the microbial load at the beginning of the fermentation consisted mainly of LAB and was high, because the coffee cherries exuded carbohydrate-rich juices, due to mechanical pressure experienced in their storage bags during the harvesting-depulping interval. The Catimor variety used in the present study appeared to have a sturdier skin and might thus be less prone to exuding juices than the Typica variety. Hence, the unavailability of carbohydrate-rich substrates could render the epiphytic microbiota (especially LAB) unable to grow significantly before the onset of the actual fermentation. However, even when this release of carbohydrates did not happen, the epiphytic LAB were able to outcompete other microbial groups during fermentation in both field experiments, likely due to the concomitant pH decrease. This means that both the duration of the harvesting-depulping interval and the coffee variety should be taken into account, when considering a possible pre-processing growth spurt of the coffee cherry epiphytic microbiota. In addition, the extent of fermentation, as reflected in the fermentation water compositions, was much lower in the present study. This was probably attributed to the colder processing environment of the location, the lower nutrient density of the fermentation water, and the relatively smaller mucilage proportion of the cherries, which were inherent to the variety used. The colder processing environment especially set limiting factors on the growth of the microbial communities. The lower counts of LAB, enterobacteria, and yeasts, as well as the absence of AAB implied a difficulty of these microorganisms to accommodate to the lower temperatures under the current experimental conditions, since they have been found in fermentations carried out in warmer regions before. Nevertheless, the fermentation duration and the processing type still played a great role in the metabolite compositions of the green coffee beans and the sensory outcomes of the coffee cups, whereas washing and soaking could lessen the fermentation effects. In addition, the presence of the endogenous bean metabolism was of influence and could modify the green coffee bean compositions through diverse reactions. Still, different processing parameters resulted in differences on the green coffee bean compositional level.

## Conclusion

The present wet coffee processing study carried out in Yunnan, China, compared the effect of demucilaging and depulping, the fermentation duration, and a soaking step on the microbial community compositions, the metabolomic profiles of the (green) coffee beans and processing waters, and the cup quality. From the microbiological perspective, the microbial composition was affected by both the processing type and fermentation duration, particularly based on the culture-independent microbial community composition. With the exception of *Lactococcus*, even represented by one ASV, all top discriminant microbial communities had a higher prevalence in fermentations of DP processes than in those of DM ones. The fermentation duration also affected the microbial community composition significantly. An extended fermentation duration increased the numerical prevalence of LAB. Of the top discriminant species between standard and extended fermentation durations, only *Lactobacillus*, represented by one ASV, was more prevalent in the fermentations of the DP processes than in those of the DM ones, even more pronounced toward the end of the extended fermentation durations, whereas all other ASVs decreased. From the metabolomic and concomitant sensory perspectives, among all the processing variants applied, the fermentation duration (impacting the microbial community composition) had the greatest impact on the green coffee bean compositions and sensory quality of the brewed coffees, followed by the processing type and the application of a soaking step. The combination of depulping and long fermentation could enhance the cup quality, especially the fruity notes of the brewed coffees. The application of soaking tempered the positive fermentation effects and standardized the green coffee bean quality, regardless of the processing practices applied. As an alternative for depulping, demucilaging could produce comparable coffee quality. However, coffees resulting from demucilaged beans were significantly less fruity in odor and had higher humus flavor. Otherwise, demucilaging tended to be more reproducible than depulping. Lastly, the impact strength of each processing parameter examined also depended on the coffee variety used and the local geographical conditions, providing a great margin of opportunities for future research. Summarizing, the present study showed that certain processing parameters need to be carefully thought through, since they will affect the microbial ecology and sensory characteristics of the brewed coffee. Complementarily, it showed that certain attributes of brewed coffee can be tweaked by changing the processing parameters.

## Data Availability Statement

The datasets generated for this study can be found in the European Nucleotide Archive of the European Bioinformatics Institute.

## Author Contributions

SZ, FD, CM, GC, and ZC conducted the experiments on the coffee plantation. FD performed the microbiological analyses. SZ performed the metabolomic analyses. VP and SW performed the bioinformatics analyses. CM coordinated the sensory analyses. SZ, FD, VP, SW, and LD wrote the manuscript. All authors read, revised, and approved the final version of the manuscript.

## Conflict of Interest

GC and CM were employed by the company Nestlé. The remaining authors declare that the research was conducted in the absence of any commercial or financial relationships that could be construed as a potential conflict of interest.

## Footnotes

1 http://www.ebi.ac.uk/ena/data/view/PRJEB30537

2 http://www.ebi.ac.uk/ena/data/view/PRJEB30538

3 http://www.ebi.ac.uk/ena/data/view/PRJEB31746

## References

[B1] Afeefy H. Y. Liebman J. F. Stein S. E. (2017). “Neutral thermochemical data,” in *NIST Chemistry WebBook, NIST Standard Reference Database Number 69*, eds Linstrom P. J. Mallard W. G. (Gaithersburg MD: National Institute of Standards and Technology), 20899 10.18434/T4D303

[B2] Altschul S. F. Gish W. Miller W. Myers E. W. Lipman D. J. (1990). Basic local alignment search tool. *J. Mol. Biol.* 215 403–410. 10.1006/jmbi.1990.9999 2231712

[B3] Bai B. Sikron N. Gendler T. Kazachkova Y. Barak S. Grafi G. (2012). Ecotypic variability in the metabolic response of seeds to diurnal hydration–dehydration cycles and its relationship to seed vigor. *Plant Cell Physiol.* 53 38–52. 10.1093/pcp/pcr169 22156384

[B4] Bates D. Maechler M. Bolker B. Walker S. (2015). Fitting linear mixed-effects models using lme4. *J. Stat. Softw.* 67:61390 10.18637/jss.v067.i01

[B5] Batista L. R. Chalfoun S. M. (2014). “Quality of coffee beans,” in *Cocoa and Coffee Fermentations*, eds Schwan R. F. Fleet G. H. (Boca Raton, FL: CRC Press), 477–508.

[B6] Bolger A. M. Lohse M. Usadel B. (2014). Trimmomatic: a flexible trimmer for Illumina sequence data. *Bioinformatics* 30 1–7. 10.1093/bioinformatics/btu170 24695404 PMC4103590

[B7] Bonilla-Hermosa V. A. Duarte F. Schwan R. F. (2014). Utilization of coffee by-products obtained from semi-washed process for production of value-added compounds. *Bioresour. Technol.* 166 142–150. 10.1016/j.biortech.2014.05.031 24907573

[B8] Borém F. M. Isquierdo E. P. da Silva Taveira J. H. (2014). “Coffee processing,” in *Handbook of Coffee Post-Harvest Technology*, ed. Borém F. M. (Norcross, GA: Gin press), 51–67.

[B9] Brando C. H. J. Brando M. F. (2014). “Methods of coffee fermentation and drying,” in *Cocoa and Coffee Fermentations*, eds Schwan R. F. Fleet G. H. (Boca Raton, FL: CRC Press), 367–396.

[B10] Bustos-Vanegas J. D. Corrêa P. C. Martins M. A. Baptestini F. M. Campos R. C. de Oliveira G. (2018). Developing predictive models for determining physical properties of coffee beans during the roasting process. *Ind. Crops Prod.* 112 839–845. 10.1016/j.indcrop.2017.12.015

[B11] Bytof G. Knopp S. E. Schieberle P. Teutsch I. Selmar D. (2005). Influence of processing on the generation of γ-aminobutyric acid in green coffee beans. *Eur. Food. Res. Technol.* 220 240–245. 10.1007/s00217-004-1033-z

[B12] Callahan B. J. McMurdie P. J. Holmes S. P. (2017). Exact sequence variants should replace operational taxonomic units in marker-gene data analysis. *ISME J.* 11 2639–2643. 10.1038/ismej.2017.119 28731476 PMC5702726

[B13] Chapagain A. K. Hoekstra A. Y. (2007). The water footprint of coffee and tea consumption in The Netherlands. *Ecol. Econ.* 64 109–118. 10.1016/j.ecolecon.2007.02.022

[B14] Cheng B. Furtado A. Smyth H. E. Henry R. J. (2016). Influence of genotype and environment on coffee quality. *Trends Food Sci. Technol.* 57 20–30. 10.1016/j.tifs.2016.09.003 28899100

[B15] De Bruyn F. Zhang J. S. Pothakos V. Torres J. Lambot C. Moroni A. V. (2017). Exploring the impacts of postharvest processing on the microbiota and metabolite profiles during green coffee bean production. *Appl. Environ. Microbiol.* 83 e2316–e2398. 10.1128/AEM.02398-16 27793826 PMC5165123

[B16] de Rosa J. S. Freitas-Silva O. Rouws J. R. C. Moreira I. G. Novaes F. J. M. Azevedo D. A. (2016). Mass spectrometry screening of Arabica coffee roasting: a non-target and non-volatile approach by EASI-MS and ESI-MS. *Food Res. Int.* 89 967–975. 10.1016/j.foodres.2016.03.021

[B17] Deibler K. D. Delwiche J. (2004). *Handbook of Flavor Characterization - Sensory Analysis, Chemistry, and Physiology.* New York, NY: Marcel Dekker.

[B18] Evangelista R. S. da Cruz M. G. Silva C. F. Pinheiro A. C. M. Schwan R. F. (2015). Microbiology diversity associated with the spontaneous wet method of coffee fermentation. *Int. J. Food Microbiol.* 210 102–112. 10.1016/j.ijfoodmicro.2015.06.008 26119187

[B19] Fait A. Fromm H. Walter D. Galili G. Fernie A. R. (2007). Highway or byway: the metabolic role of the GABA shunt in plants. *Trends Plant Sci.* 13 14–19. 10.1016/j.tplants.2007.10.005 18155636

[B20] Farooq M. Wahid A. Kobayashi N. Fujita D. Basra S. M. A. (2009). Plant drought stress: effects, mechanisms and management. *Agron. Sustain. Dev.* 29 185–212. 10.1051/agro:2008021

[B21] Feng X. Dong H. Yang P. Yang R. Lu J. Lv J. (2016). Culture-dependent and -independent methods to investigate the predominant microorganisms associated with wet processed coffee. *Curr. Microbiol.* 73 190–195. 27113591 10.1007/s00284-016-1047-3

[B22] Fernández M. Zúñiga M. (2006). Amino acid catabolic pathways of lactic acid bacteria. *Crit. Rev. Microbiol.* 32 155–183. 10.1080/10408410600880643 16893752

[B23] Gänzle M. G. (2015). Lactic metabolism revisited: metabolism of lactic acid bacteria in food fermentations and food spoilage. *Curr. Opin. Food Sci.* 2 106–117. 10.1016/j.cofs.2015.03.001

[B24] Gloess A. N. Yeretzian C. Knochenmuss R. Groessi M. (2018). On-line analysis of coffee roasting with ion mobility spectrometry-mass spectrometry (IMS-MS). *Int. J. Mass. Spectrom.* 424 49–57. 10.1016/j.ijms.2017.11.017

[B25] Gonzalez-Rios O. Suarez-Quiroz M. L. Boulanger R. Barel M. Guyot B. Guiraud J. P. (2007). Impact of “ecological” post-harvest processing on the volatile fraction of coffee beans: I. *Green coffee. J. Food Compost. Anal.* 20 289–296. 10.1016/j.jfca.2006.07.009

[B26] Hamdouche Y. Meile J. C. Nganou D. N. Durand N. Teyssier C. Montet D. (2016). Discrimination of post-harvest coffee processing methods by microbial ecology analyses. *Food Control* 65 112–120. 10.1016/j.foodcont.2016.01.022

[B27] Harrell F. E. (2018). *Hmisc: Harrell Miscellaneous.* Available at: https://cran.r-project.org/web/packages/Hmisc/Hmisc.pdf (accessed March, 2018).

[B28] Häusler R. E. Ludewig F. Krueger S. (2014). Amino acids - a life between metabolism and signaling. *Plant Sci.* 229 225–237. 10.1016/j.plantsci.2014.09.011 25443849

[B29] Hildebrandt T. M. Nesi A. N. Araújo W. L. Braun H. P. (2015). Amino acid catabolism in plants. *Mol. Plant* 8 1563–1579. 10.1016/j.molp.2015.09.00 26384576

[B30] Kim W. (2014). “The genus *Lactococcus*,” in *Lactic Acid Bacteria: Biodiversity and Taxonomy*, eds Holzapfel W. H. Wood B. J. B. (Weinheim: Wiley-VCH Verlag), 429–444.

[B31] Kramer D. Breitenstein B. Kleinwächter M. Selmar D. (2010). Stress metabolism in green coffee beans (*Coffea arabica* L.): expression of dehydrins and accumulation of GABA during drying. *Plant Cell Phys.* 4 546–553. 10.1093/pcp/pcq019 20208063

[B32] Labbe D. Sudre J. Dugas V. Folmer B. (2016). Impact of crema on expected and actual espresso coffee experience. *Food Res. Int.* 82 53–58. 10.1016/j.foodres.2016.01.020

[B33] Lambot C. Herrera J. C. Bertrand B. Sadeghian S. Benavides P. Gaitán A. (2017). “Cultivating coffee quality - terroir and agro-ecosystem,” in *The Craft and Science of Coffee*, ed. Folmer B. (Cambridge, MA: Academic Press), 17–49. 10.1016/b978-0-12-803520-7.00002-5

[B34] Laureys D. De Vuyst L. (2014). Microbial species diversity, community dynamics, and metabolite kinetics of water kefir fermentation. *Appl. Environ. Microbiol.* 80 2564–2572. 10.1128/AEM.03978-13 24532061 PMC3993195

[B35] Lee L. W. Cheong M. W. Curran P. Yu B. Liu S. Q. (2015). Coffee fermentation and flavor - an intricate and delicate relationship. *Food Chem.* 185 182–191. 10.1016/j.foodchem.2015.03.124 25952856

[B36] Li D. H. Liu C. M. Luo R. B. Sadakane K. Lam T. W. (2015). MEGAHIT: an ultra-fast single-node solution for large and complex metagenomics assembly via succinct de Bruijn graph. *Bioinformatics* 31 1674–1676. 10.1093/bioinformatics/btv033 25609793

[B37] Limami A. M. Glévarex G. Ricoult C. Cliquet J. B. Planchet E. (2008). Concerted modulation of alanine and glutamate metabolism in young *Medicago truncatula* seedlings under hypoxic stress. *J. Exp. Bot.* 59 2325–2335. 10.1093/jxb/ern102 18508812 PMC2423662

[B38] Masella A. P. Bartram A. K. Truszkowski J. M. Brown D. G. Neufeld J. D. (2012). PANDAseq: paired-end assembler for Illumina sequences. *BMC Bioinformatics* 13:31. 10.1186/1471-2105 22333067 PMC3471323

[B39] Mosciano G. (2018). *Flavor Library.* Available at: https://www.perfumerflavorist.com/flavor/library/ (accessed March, 2018).

[B40] Murthy P. S. Naidu M. M. (2012). Sustainable management of coffee industry by-products and value addition - a review. *Resour. Conserv. Recycl.* 66 45–58. 10.1016/j.resconrec.2012.06.005

[B41] Neu A. K. Pleissner D. Mahlmann K. Schneider R. Puerta-Quintero G. Venus J. (2016). Fermentative utilization of coffee mucilage using *Bacillus coagulans* and investigation of down-stream processing of fermentation broth for optically pure L(+)-lactic acid production. *Bioresour. Technol.* 211 398–405. 10.1016/j.biortech.2016.03.122 27035470

[B42] Oksanen J. Blanchet G. F. Friendly M. Kindt R. Legendre P. (2018). *vegan: Community Ecology Package.* Available at: https://cran.r-project.org/web/packages/vegan/vegan.pdf (accessed March, 2018).

[B43] Papalexandratou Z. Lefeber T. Ong Seng, Lee B. B. Daniel H. M. De Vuyst L. (2013). *Hanseniaspora opuntiae, Saccharomyces cerevisiae, Lactobacillus fermentum, and Acetobacter pasteurianus* predominate during well-performed Malaysian cocoa bean box fermentations, underlining the importance of these microbial species for a successful cocoa bean fermentation process. *Food Microbiol.* 35 73–85. 10.1016/j.fm.2013.02.015 23664257

[B44] Parenti A. Guerrini L. Masella P. Spinelli S. Calamai L. Spugnoli P. (2014). Comparison of espresso coffee brewing techniques. *J. Food Eng.* 121 112–117. 10.1016/j.jfoodeng.2013.08.031

[B45] Pereira G. V. Carvalho Neto D. P. Magalhães A. I. Vásquez Z. S. Medeiros A. B. P. Vandenberghe L. P. S. (2019). Exploring the impacts of post-harvest processing on the aroma formation of coffee beans: a review. *Food Chem.* 272 441–452. 10.1016/j.foodchem.2018.08.061 30309567

[B46] Pereira G. V. Soccol V. T. Brar S. K. Neto E. Soccol C. R. (2017). Microbial ecology and starter culture technology in coffee processing. *Crit. Rev. Food Sci. Nutr.* 57 2775–2788. 10.1080/10408398.2015.1067759 26462969

[B47] Poisson L. Blank I. Dunkel A. Hofmann T. (2017). “Chapter 12 the chemistry of roasting-decoding flavour formation,” in *The Craft and Science of Coffee*, ed. Folmer B. (Cambdrige, MA: Academic Press), 273–309. 10.1016/b978-0-12-803520-7.00012-8

[B48] Pot B. Felis G. E. De Bruyne K. Tsakalidou E. Papadimitriou K. Leisner J. (2014). “The genus *Lactobacillus*,” in *Lactic Acid Bacteria: Biodiversity and Taxonomy*, eds Holzapfel W. H. Wood B. J. B. (Weinheim: Wiley-VCH Verlag), 249–354.

[B49] Pothakos V. Snauwaert C. De Vos P. Huys G. Devlieghere F. (2014). Monitoring psychrotrophic lactic acid bacteria contamination in a ready-to-eat vegetable salad production environment. *Int. J. Food Microbiol.* 185 7–16. 10.1016/j.ijfoodmicro.2014.05.009 24927398

[B50] R Core Team (2018). *R: A Language and Environment for Statistical Computing.* Available at: https://www.gbif.org/tool/81287/r-a-language-and-environment-for-statistical-computing (accessed March, 2018).

[B51] Rendón M. Y. Gratão P. L. Salva T. J. Azevedo R. A. Bragagnolo N. (2013). Antioxidant enzyme activity and hydrogen peroxide content during the drying of *arabica* coffee beans. *Eur. Food. Res. Technol.* 236 753–758. 10.1007/s00217-013-1933-x

[B52] Sakiyama N. S. Ferrão M. A. (2014). “Botany and production of coffee,” in *Cocoa and Coffee Fermentations*, Schwan R. F. Fleet G. H. eds (Boca Raton, FL: CRC Press), 341–361.

[B53] Sanz-Uribe J. R. Yusianto, Menon S. N. Peñuela A. Oliveros C. Husson J. (2017). “Postharvest processing - revealing the green bean,” in *The Craft and Science of Coffee*, ed. Folmer B. (Cambridge, MA: Academic Press), 51–79. 10.1016/b978-0-12-803520-7.00003-7

[B54] Sato K. Yamane M. Yamaji N. Kanamori H. Tagiri A. Schwerdt J. G. (2016). Alanine aminotransferase controls seed dormancy in barley. *Nat. Commun.* 7:11625. 10.1038/ncomms11625 27188711 PMC4873977

[B55] Seemann T. (2014). Prokka: rapid prokaryotic genome annotation. *Bioinformatics* 30 2068–2069. 10.1093/bioinformatics/btu153 24642063

[B56] Selmar D. Bytof G. Knopp S. E. Breitenstein B. (2006). Germination of coffee seeds and its significance for coffee quality. *Plant Biol.* 8 260–264. 10.1055/s-2006-923845 16547871

[B57] Silva C. F. (2014). “Microbial activity during coffee fermentation,” in *Cocoa and Coffee Fermentations*, eds Schwan R. F. Fleet G. H. (Boca Raton, FL: CRC Press), 398–423.

[B58] Silva C. F. Batista L. R. Abreu L. M. Dias E. S. Schwan R. F. (2008). Succession of bacterial and fungal communities during natural coffee (*Coffea arabica*) fermentation. *Food Microbiol.* 25 951–957. 10.1016/j.fm.2008.07.003 18954729

[B59] Snowden C. J. Thomas B. Baxter C. J. Smith J. A. C. Sweetlove L. J. (2015). A tonoplast Glu/Asp/GABA exchanger that affects tomato fruit amino acid composition. *Plant J.* 81 651–660. 10.1111/tpj.12766 25602029 PMC4950293

[B60] Vaughan M. J. Mitchell T. McSpadden Gardener B. B. (2015). What’s inside that seed we brew? a new approach to mining the coffee microbiome. *Appl. Environ. Microbiol.* 81 6518–6552. 10.1128/AEM.01933-15 26162877 PMC4561686

[B61] Velmourougane K. (2011). Effects of wet processing methods and subsequent soaking of coffee under different organic acids on cup quality. *World J. Sci. Technol.* 1 32–38.

[B62] Vermote L. Verce M. De Vuyst L. Weckx S. (2018). Amplicon and shotgun metagenomic sequencing indicates that microbial ecosystems present in cheese brines reflect environmental inoculation during the cheese production process. *Int. Dairy J.* 87 44–53. 10.1016/j.idairyj.2018.07.010

[B63] Waters D. M. Moroni A. V. Arendt E. K. (2017). Overview on the mechanisms of coffee germination and fermentation and their significance for coffee and coffee beverage quality. *Crit. Rev. Food Sci. Nutr.* 57 259–274. 10.1080/10408398.2014.902804 26020134

[B64] Wei T. Simko V. (2017). *corrplot: Visualization of a Correlation Matrix.* Available at: https://cran.r-project.org/web/packages/corrplot/corrplot.pdf (accessed March, 2018).

[B65] Wickham H. (2016). *ggplot2: Create Elegant Data Visualisations Using the Grammar of Graphics.* Available at: https://cran.r-project.org/web/packages/ggplot2/ggplot2.pdf (accessed March, 2018).

[B66] Wickham H. (2019). *Gplots: Various R Programming Tools for Plotting Data.* Available at: https://cran.r-project.org/web/packages/gplots/gplots.pdf (accessed January, 2019).

[B67] Yilmaz B. Acar-Tek N. Sözlü S. (2017). Turkish cultural heritage: a cup of coffee. *J. Ethn. Foods* 4 213–220. 10.1016/j.jef.2017.11.003

[B68] Zaunmüller T. Eichert M. Richter H. Unden G. (2006). Variations in the energy metabolism of biotechnologically relevant heterofermentative lactic acid bacteria during growth on sugars and organic acids. *Appl. Microbiol. Biotechnol.* 72 421–429. 10.1007/s00253-006-0514-3 16826375

[B69] Zhang J. S. De Bruyn F. Pothakos V. Torres J. Moccand C. Weckx S. (2019). Following coffee production from cherries to cup: microbiological and metabolomic analysis of wet processing of coffea *arabica*. *Appl. Environ. Microbiol.* 85:e02635-18. 10.1128/AEM.02635-18 30709820 PMC6414394

