# Supplementary material for: Influence of Various Processing Parameters on the Microbial Community Dynamics, Metabolomic Profiles, and Cup Quality During Wet Coffee Processing
Source: Front Microbiol. 2019 Nov 13;10:2621. doi: 10.3389/fmicb.2019.02621 (PMC6863779; doi:10.3389/fmicb.2019.02621)
Supplement: Supplementary file 1 [file Data_Sheet_1.PDF]

Supplementary Material

Supplementary Table 1. Overview of the number of occasions a known protein was annotated with an EC number belonging to the sub-subclasses indicated, after sequencing of a fermentation sample from the Arabica coffee wet processing experiments, using a shotgun metagenomics approach.

EC sub-subclass	DM1_F72	DM2_F72	DP1_F36	DP1_F84	DP2_F48	DP2_F84
1.1.1.-	567	541	874	1641	1125	1326
3.6.3.-	602	659	859	1401	1135	1127
2.7.1.-	551	525	906	1482	1126	1145
2.1.1.-	542	587	828	1285	976	1062
2.7.7.-	428	478	688	1072	856	937
2.3.1.-	435	451	672	1060	853	919
2.4.1.-	506	522	709	951	790	745
3.2.1.-	342	412	522	881	676	766
6.1.1.-	327	365	469	723	636	649
3.1.3.-	280	293	465	811	634	625
3.6.4.-	285	282	338	558	461	492
2.5.1.-	223	218	382	580	449	491
4.2.1.-	200	207	305	662	434	489
3.6.1.-	208	228	354	520	432	469
3.5.1.-	195	175	322	488	443	440
2.4.2.-	181	181	280	359	334	356
3.4.21.-	180	167	259	384	340	289
2.7.13.-	134	152	227	407	313	310
2.6.1.-	147	143	237	405	278	327
5.1.3.-	136	145	201	393	262	285
2.7.8.-	140	159	243	338	247	276
5.3.1.-	136	167	187	337	263	274
6.3.4.-	130	148	234	289	252	292
4.1.1.-	115	114	175	375	308	235
6.3.2.-	130	132	189	312	231	239
5.4.99.-	138	114	174	280	223	254
6.3.5.-	128	114	181	249	206	260
3.1.1.-	101	85	147	284	225	261
2.7.4.-	123	128	164	215	199	228
3.1.4.-	62	71	119	258	230	218

Table 1 *(Continued)*

EC sub-subclass	DM1_F72	DM2_F72	DP1_F36	DP1_F84	DP2_F48	DP2_F84
5.4.2.-	94	87	145	236	186	208
3.1.26.-	91	109	138	203	195	189
3.2.2.-	89	84	123	236	186	193
1.3.1.-	78	84	121	233	168	200
3.4.24.-	98	83	131	200	164	163
3.4.11.-	69	94	134	189	167	173
5.99.1.-	68	96	121	191	161	171
1.8.1.-	83	98	131	198	132	158
1.2.1.-	60	66	115	187	179	184
3.5.4.-	76	86	119	187	130	184
2.8.1.-	49	57	132	238	159	139
2.7.2.-	61	71	91	202	151	184
2.2.1.-	52	76	114	210	134	145
4.1.2.-	52	61	97	218	145	157
1.5.1.-	52	74	97	172	133	154
2.7.6.-	53	51	103	143	107	135
3.1.21.-	75	92	90	122	105	104
2.7.11.-	47	63	98	137	102	101
5.2.1.-	69	59	87	136	116	81
5.1.1.-	43	48	82	132	110	126
1.11.1.-	41	46	77	139	112	119
1.17.4.-	70	69	85	119	79	100
1.6.5.-	27	40	66	145	133	110
6.3.1.-	40	52	63	142	101	104
1.8.4.-	61	52	73	105	81	100
3.1.11.-	42	35	70	147	69	94
4.3.1.-	34	41	63	124	92	103
4.4.1.-	32	48	51	119	83	108
4.1.3.-	39	38	62	98	87	86
1.6.99.-	33	30	54	104	86	97
3.4.13.-	39	40	72	90	66	87
2.1.2.-	39	37	76	89	76	75
4.2.99.-	37	27	64	84	86	86
6.4.1.-	32	35	51	96	69	72
4.2.3.-	39	36	49	92	64	73
3.5.99.-	36	36	54	91	59	70
6.5.1.-	43	53	60	59	70	54

Table 1 *(Continued)*

EC sub-subclass	DM1_F72	DM2_F72	DP1_F36	DP1_F84	DP2_F48	DP2_F84
2.1.3.-	34	24	64	92	48	69
6.2.1.-	57	50	29	65	64	65
3.6.5.-	35	35	46	76	58	68
3.4.22.-	27	44	55	80	43	65
2.3.2.-	49	36	43	78	47	46
3.5.2.-	24	30	45	82	61	51
6.3.3.-	33	32	42	67	47	49
4.3.2.-	29	22	36	67	50	55
4.2.2.-	23	12	38	78	52	46
1.10.3.-	22	22	32	62	42	56
2.7.10.-	21	34	35	63	39	42
3.1.2.-	12	16	28	67	59	49
1.97.1.-	14	21	42	57	39	57
3.4.16.-	25	19	30	50	50	52
2.4.99.-	27	29	34	55	37	40
1.2.4.-	27	29	36	44	47	38
2.3.3.-	28	20	33	48	39	53
1.3.99.-	20	17	27	81	33	36
1.13.11.-	24	25	33	44	46	28
1.18.1.-	19	24	33	41	34	37
1.3.5.-	6	12	33	48	47	41
3.1.27.-	18	18	21	46	38	40
1.4.1.-	21	14	27	55	34	25
4.3.3.-	15	11	19	32	40	43
5.3.3.-	13	18	23	40	34	28
1.1.98.-	14	6	24	45	29	32
2.7.3.-	21	14	19	41	17	38
1.20.4.-	15	11	21	41	29	32
3.5.3.-	12	17	24	40	30	24
1.17.1.-	19	16	23	39	27	22
3.1.22.-	14	14	19	37	25	31
1.7.1.-	5	14	23	47	27	21
3.4.14.-	26	19	17	29	21	25
4.1.99.-	6	8	12	49	36	26
3.1.13.-	15	12	12	30	29	34
1.14.13.-	6	0	24	48	40	12
3.1.30.-	22	19	26	22	18	22
1.2.3.-	14	11	21	25	27	29
1.1.5.-	2	1	24	39	32	23
1.15.1.-	4	7	14	43	32	20

Table 1 *(Continued)*

EC sub-subclass	DM1_F72	DM2_F72	DP1_F36	DP1_F84	DP2_F48	DP2_F84
5.3.2.-	5	12	18	28	21	26
1.14.14.-	10	10	10	34	18	26
5.4.4.-	8	11	11	20	27	23
3.7.1.-	8	12	13	26	26	12
3.4.23.-	8	3	11	32	17	18
1.2.7.-	8	6	19	20	18	5
1.7.99.-	0	0	9	25	21	21
1.13.12.-	9	9	15	19	8	14
1.5.3.-	8	7	8	24	15	10
3.4.19.-	6	8	10	20	6	22
1.17.99.-	5	5	7	23	17	8
4.99.1.-	3	4	8	18	12	18
1.1.3.-	3	3	10	16	12	17
1.3.98.-	12	10	6	12	11	8
1.4.3.-	1	2	9	20	14	9
1.16.3.-	0	0	12	24	15	3
1.6.1.-	0	3	6	11	18	15
1.17.7.-	4	4	13	13	11	6
2.8.2.-	3	3	6	13	15	10
1.14.11.-	0	0	12	15	9	13
4.3.99.-	0	3	6	13	17	10
5.4.3.-	3	0	9	16	9	12
2.7.9.-	0	0	8	15	15	10
2.9.1.-	2	2	9	21	11	3
3.4.17.-	7	9	3	11	13	5
2.8.4.-	6	3	6	14	6	9
1.8.5.-	0	0	3	15	15	9
4.6.1.-	5	5	8	11	11	2
1.4.7.-	2	10	11	5	5	6
1.14.12.-	2	2	8	4	7	14
1.9.3.-	6	3	22	6	0	0
2.8.3.-	0	0	6	9	3	17
3.1.6.-	0	0	0	9	12	14
1.3.8.	4	0	6	6	9	9
3.8.1.-	0	2	3	8	9	10
1.4.4.-	0	3	3	12	3	9
3.1.5.-	0	0	9	12	6	3
3.3.2.-	5	5	7	7	0	3

Table 1 *(Continued)*

EC sub-subclass	DM1_F72	DM2_F72	DP1_F36	DP1_F84	DP2_F48	DP2_F84
1.7.2.-	0	0	6	6	6	6
3.4.25.-	0	0	6	3	6	9
3.1.31.-	2	0	3	10	2	2
3.4.15.-	0	0	6	3	6	3
5.3.99.-	0	0	3	6	3	4
1.2.5.-	3	0	3	0	6	3
1.4.99.-	0	0	3	6	3	3
3.9.1.-	0	0	0	9	3	3
5.5.1.-	0	0	0	6	6	3
1.12.1.-	0	0	4	7	3	0
1.21.4.-	0	0	3	6	0	3
1.3.3.-	0	0	0	3	3	6
2.1.4.-	3	3	3	1	1	1
2.10.1.	0	0	3	3	3	3
5.1.99.-	3	0	0	3	3	3
3.3.1.-	0	0	2	3	3	3
1.21.98.-	3	0	0	0	1	3
1.8.98.-	1	0	1	1	1	3
1.12.99.-	0	0	0	6	0	0
1.14.99.-	0	0	0	6	0	0
1.18.6.-	0	0	0	0	3	3
1.8.99.-	0	0	0	6	0	0
3.11.1.-	0	0	0	0	6	0
4.7.1.-	0	0	0	3	3	0
1.17.98.-	0	0	0	3	0	0
1.2.99.-	0	0	0	0	3	0
1.3.7.-	0	0	0	3	0	0
1.5.99.-	0	0	0	0	3	0
2.6.99.-	0	0	0	0	3	0
1.12.7.-	0	0	1	0	0	0
1.8.7.-	0	0	0	0	1	0

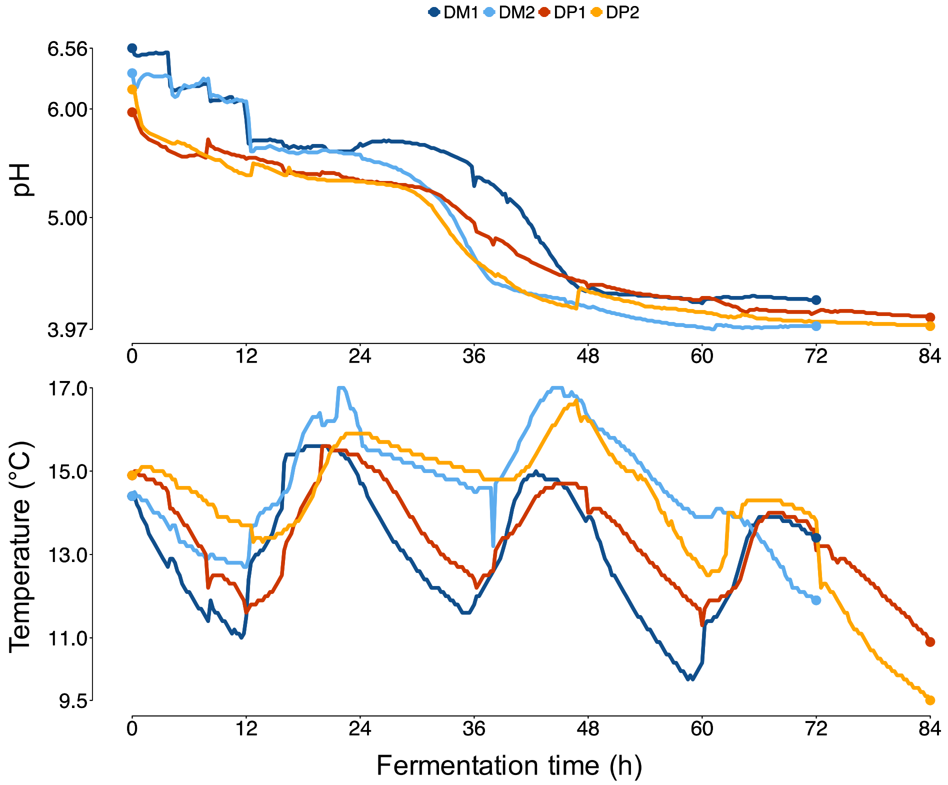

Supplementary Figure 1. On-line monitoring of pH (top) and temperature (bottom) of the fermentation masses of the depulped (DP1 and DP2) and demucilaged (DM1 and DM2) Arabica coffee wet processing experiments.

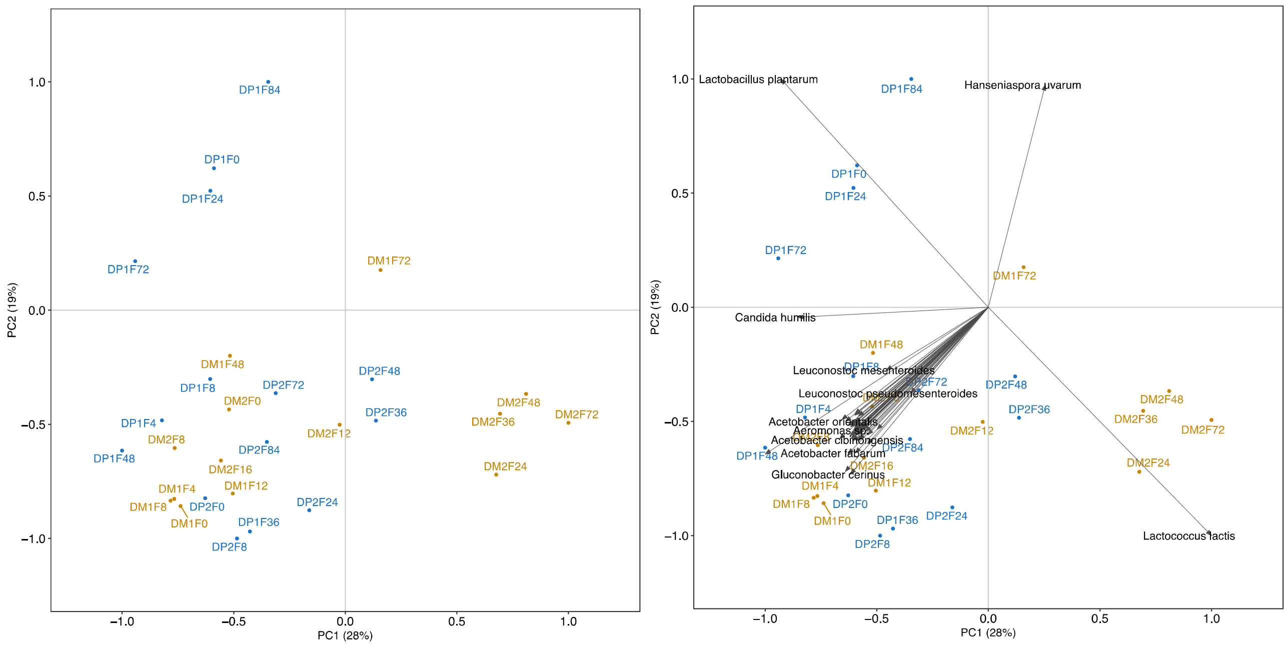

Supplementary Figure 2. Principal component analysis (PCA) of the isolate identification data without (left) and with factor loadings (right) of the Arabica coffee wet processing experiments. Samples originating from the fermentations of the depulped processes (DP) are shown in blue; fermentation samples of the demucilaged processes (DM) are shown in yellow.

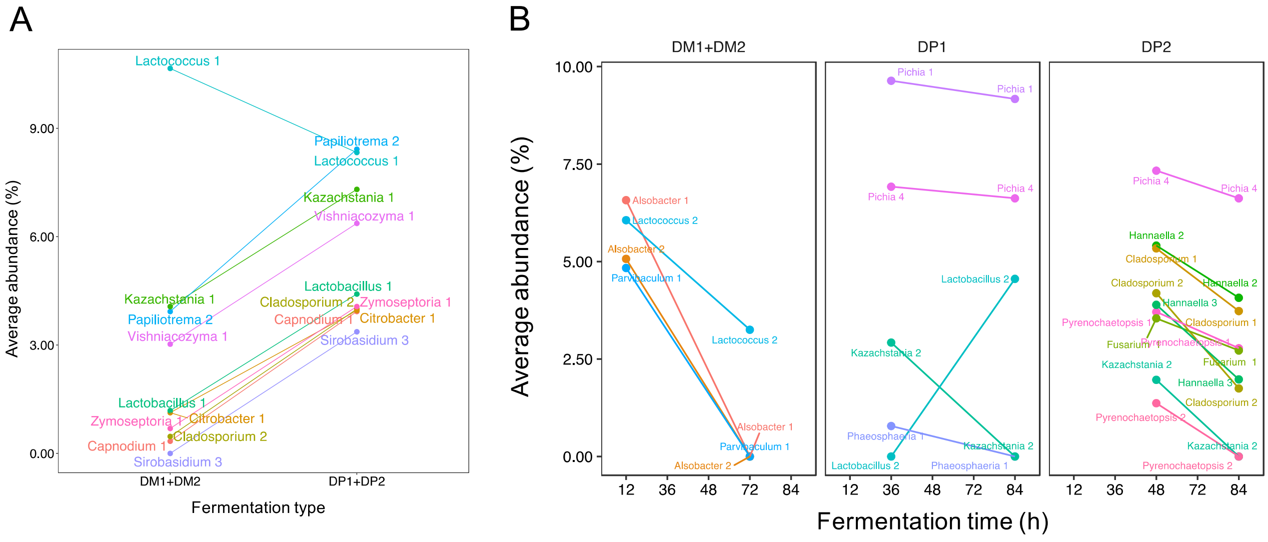

Supplementary Figure 3. Effect of demucilaging and fermentation duration on the microbial and fungal amplicon sequence variants (ASVs) of the Arabica coffee wet processing experiments. (A) Top ten discriminant amplicon ASVs of the fermentations of the demucilaged processes (DM1+DM2) and those of the fermentations of the depulped processes (DP1+DP2), ordered according to their average relative abundances in each fermentation variant. (B) Significantly discriminant ASVs between standard and extended fermentation durations for the demucilaged (DM) and depulped (DP) coffee beans.

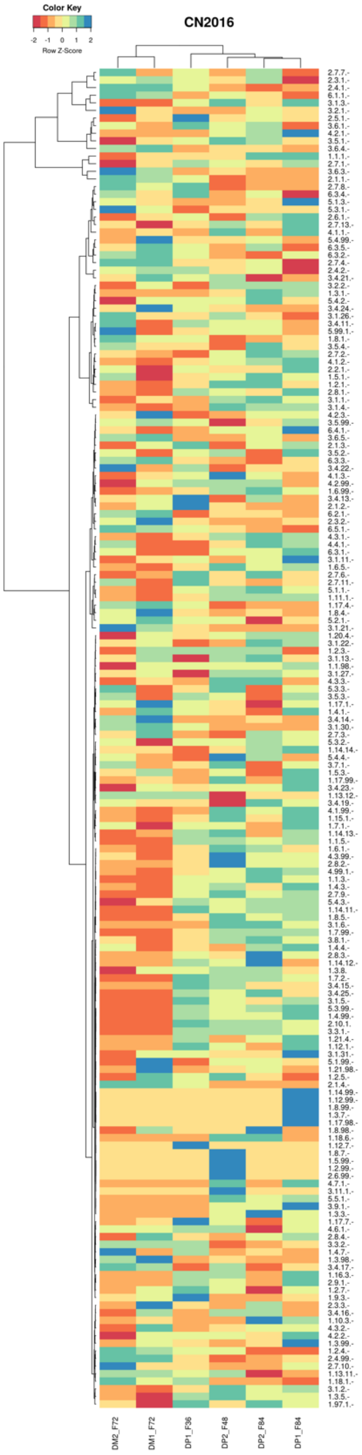

Supplementary Figure 4. Heatmap visualization of the over- and under-representation of each of the 176 enzyme sub-subclasses in the six fermentation samples of the Arabica coffee wet processing experiments, which were subjected to shotgun metagenomic sequencing, scaled per sub-subclass and represented by a Z-score.

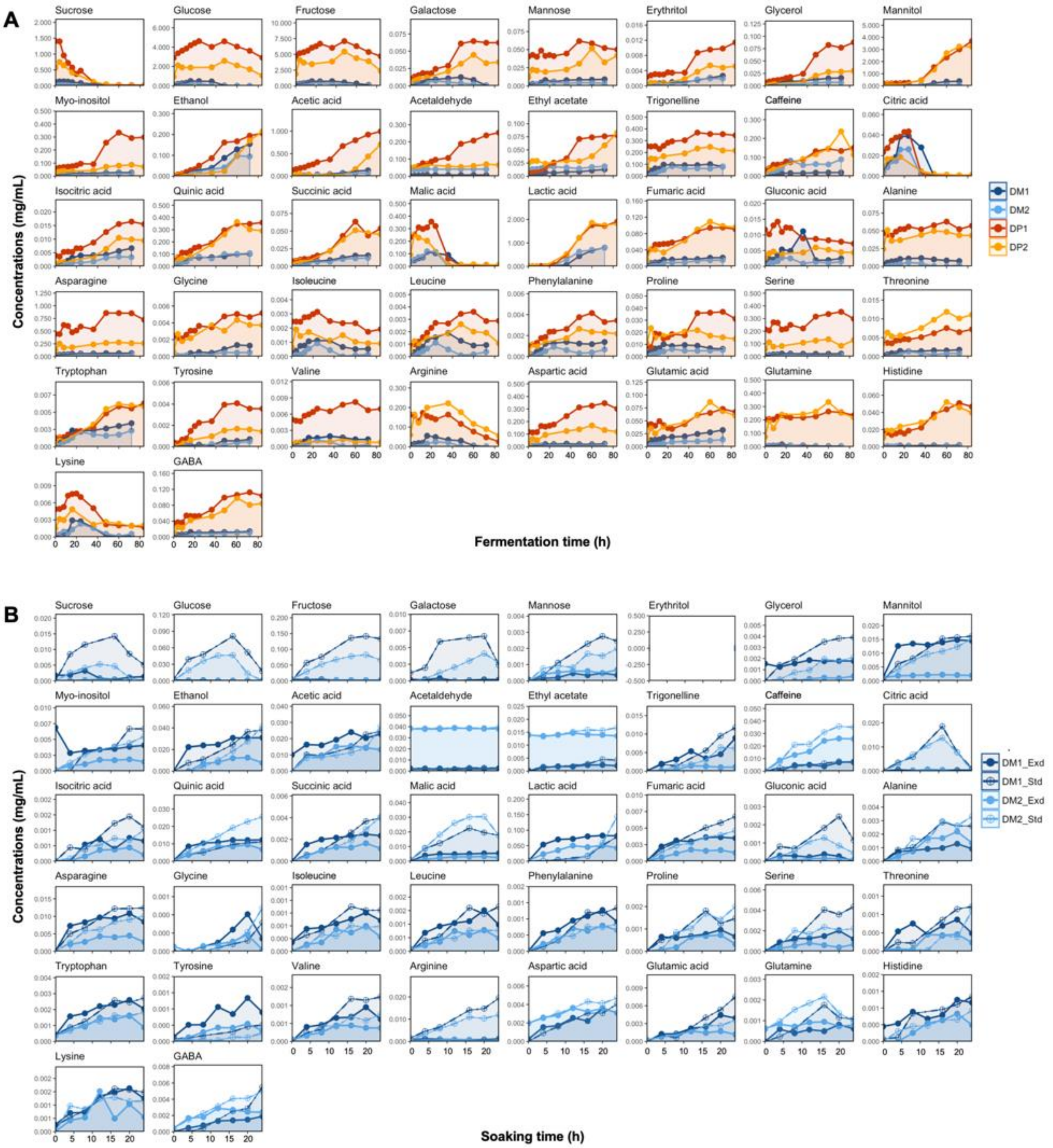
A
Sucrose
Glucose
Fructose
Galactose
Mannose
Erythritol
Glycerol
Mannitol
Myo-inositol
Ethanol
Acetic acid
Acetaldehyde
Ethyl acetate
Trigonelline
Caffeine
Citric acid
Isocitric acid
Quinic acid
Succinic acid
Malic acid
Lactic acid
Fumaric acid
Gluconic acid
Alanine
Asparagine
Glycine
Isoleucine
Leucine
Phenylalanine
Proline
Serine
Threonine
Tryptophan
Tyrosine
Valine
Arginine
Aspartic acid
Glutamic acid
Glutamine
Histidine
Lysine
GABA
DM1
DM2
DP1
DP2
Concentrations (mg/mL)
Fermentation time (h)
B
Sucrose
Glucose
Fructose
Galactose
Mannose
Erythritol
Glycerol
Mannitol
Myo-inositol
Ethanol
Acetic acid
Acetaldehyde
Ethyl acetate
Trigonelline
Caffeine
Citric acid
Isocitric acid
Quinic acid
Succinic acid
Malic acid
Lactic acid
Fumaric acid
Gluconic acid
Alanine
Asparagine
Glycine
Isoleucine
Leucine
Phenylalanine
Proline
Serine
Threonine
Tryptophan
Tyrosine
Valine
Arginine
Aspartic acid
Glutamic acid
Glutamine
Histidine
Lysine
GABA
DM1_Exd
DM1_Std
DM2_Exd
DM2_Std
Concentrations (mg/mL)
Soaking time (h)

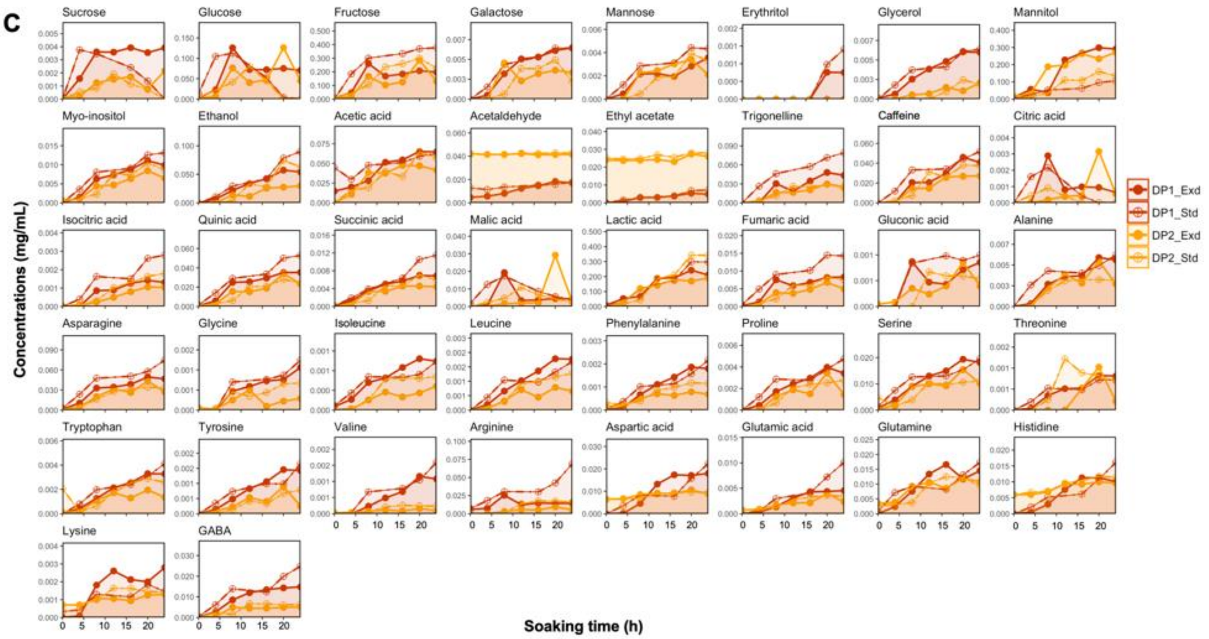

Supplementary Figure 5. Dynamics of the chemical profiles (in mg/mL) of the processing waters during fermentation (A), soaking of the demucilaged processes (B), and soaking of the depulped processes (C) of the Arabica coffee wet processing experiments.

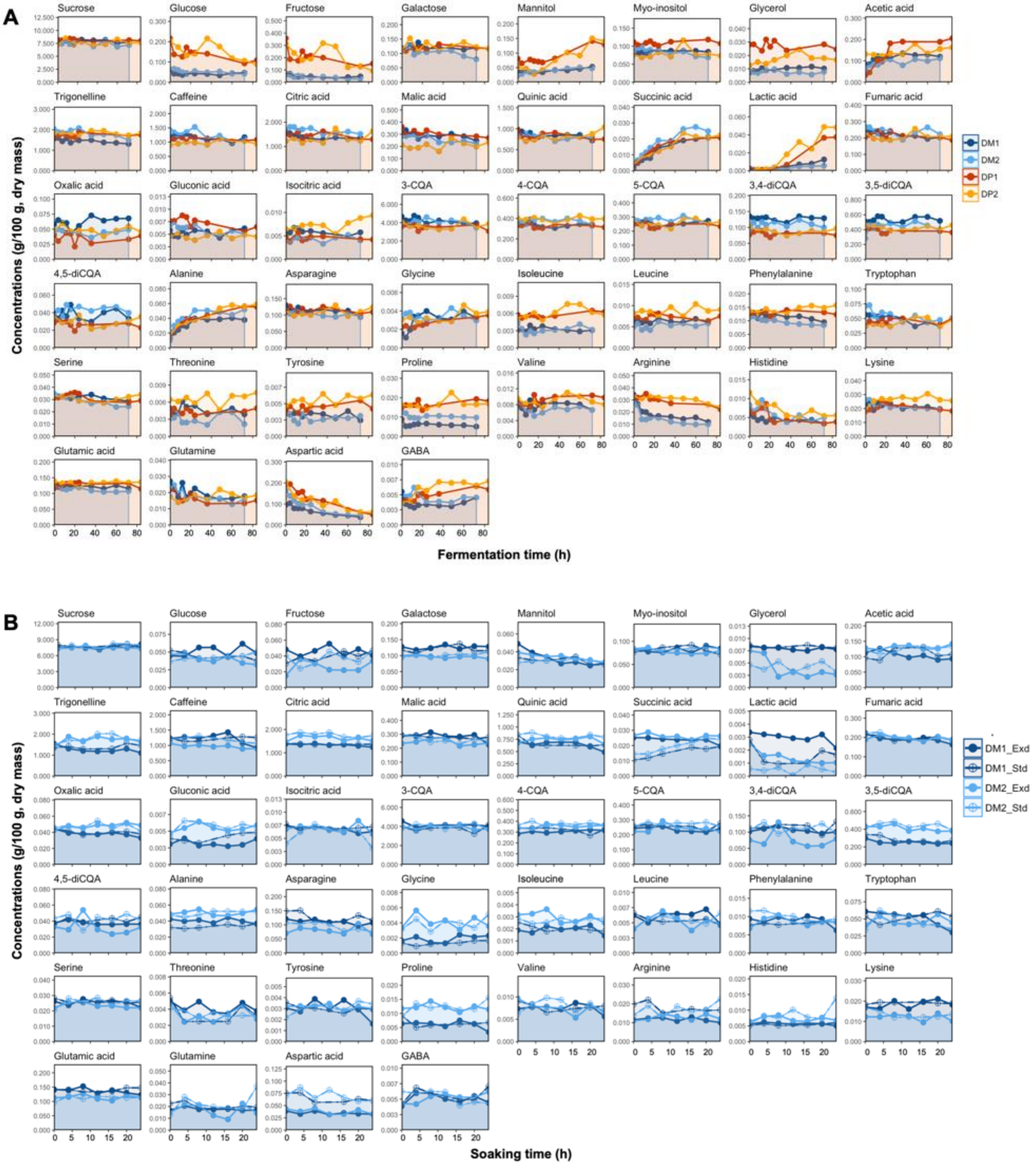
A
Sucrose
Glucose
Fructose
Galactose
Mannitol
Myo-inositol
Glycerol
Acetic acid
Trigonelline
Caffeine
Citric acid
Malic acid
Quinic acid
Succinic acid
Lactic acid
Fumaric acid
Oxalic acid
Gluconic acid
Isocitric acid
3-CQA
4-CQA
5-CQA
3,4-diCQA
3,5-diCQA
4,5-diCQA
Alanine
Asparagine
Glycine
Isoleucine
Leucine
Phenylalanine
Tryptophan
Serine
Threonine
Tyrosine
Proline
Valine
Arginine
Histidine
Lysine
Glutamic acid
Glutamine
Aspartic acid
GABA
DM1
DM2
DP1
DP2
Concentrations (g/100 g, dry mass)
Fermentation time (h)
B
DM1_Exd
DM1_Std
DM2_Exd
DM2_Std
Concentrations (g/100 g, dry mass)
Soaking time (h)

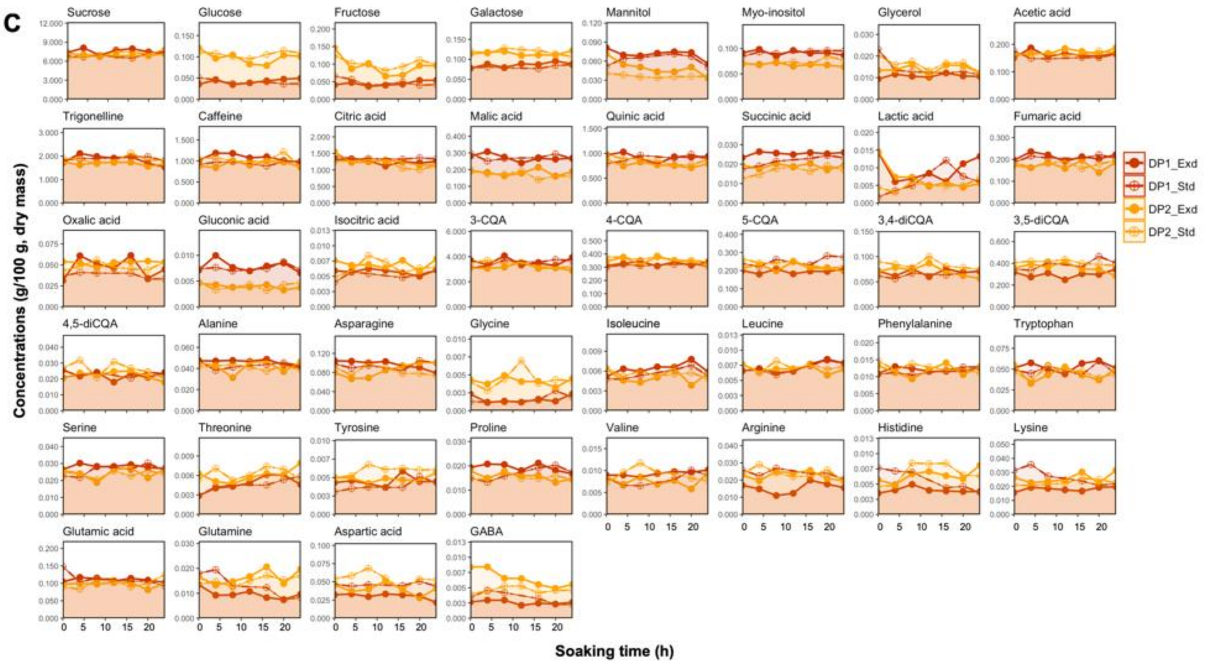

Supplementary Figure 6. Dynamics of the chemical profiles (in g/100 g, dry mass) of the coffee beans during fermentation (A), soaking of the demucilaged processes (B), and soaking of the depulped processes (C) of the Arabica coffee wet processing experiments.

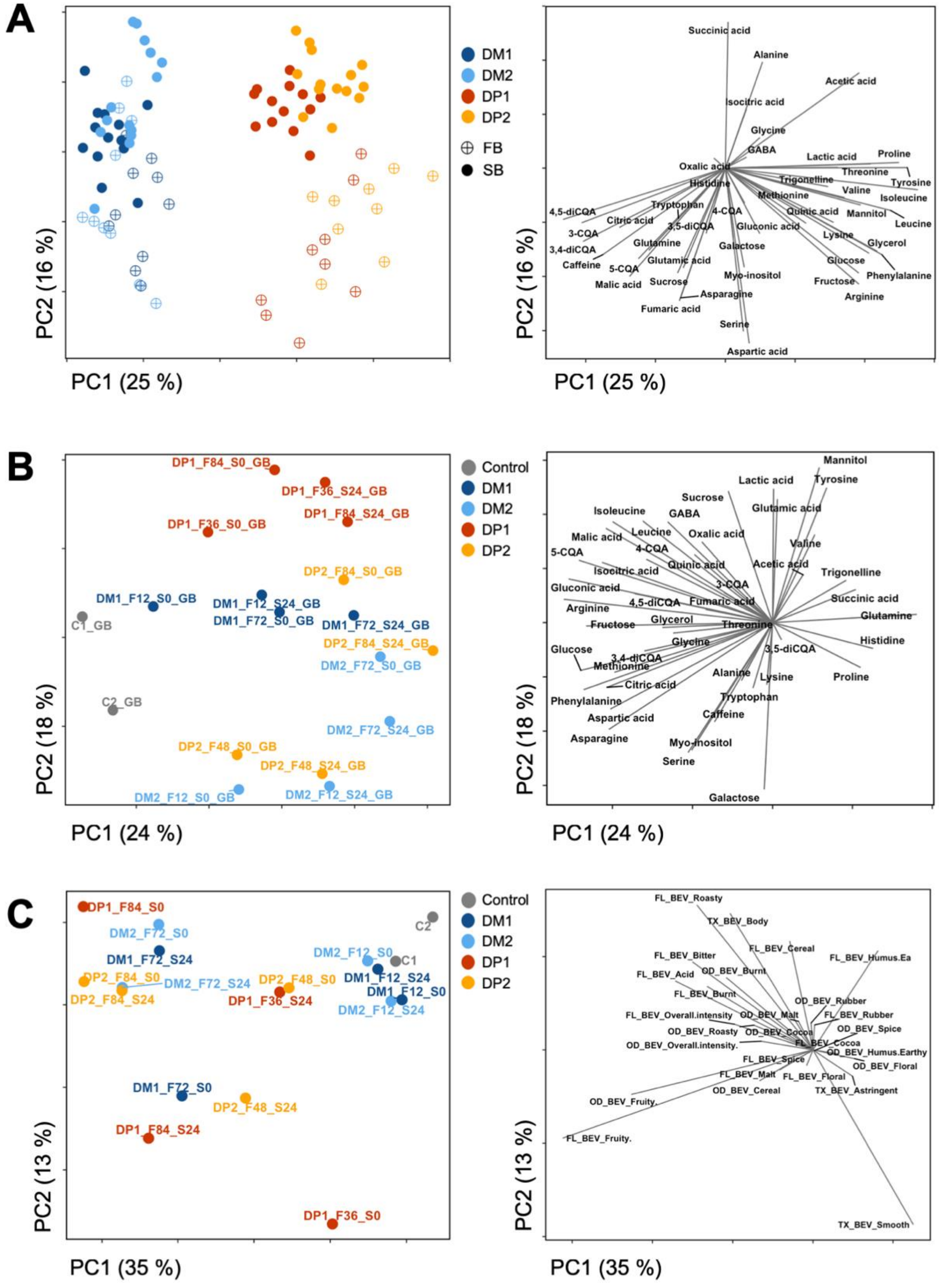
A
DM1
DM2
DP1
DP2
FB
SB
PC2 (16 %)
PC1 (25 %)
Succinic acid
Alanine
Acetic acid
Isocitric acid
Glycine
GABA
Lactic acid
Proline
Oxalic acid
Histidine
Trigonelline
Threonine
Tyrosine
Valine
Isoleucine
Methionine
Tryptophan
4-CQA
Quinic acid
Mannitol
Leucine
4,5-diCQA
Citric acid
3,5-diCQA
Gluconic acid
Lysine
Glycerol
3-CQA
Glutamine
Galactose
3,4-diCQA
Caffeine
5-CQA
Glutamic acid
Glucose
Phenylalanine
Malic acid
Sucrose
Myo-inositol
Fructose
Asparagine
Arginine
Fumaric acid
Serine
Aspartic acid
B
Control
DM1
DM2
DP1
DP2
DP1_F84_S0_GB
DP1_F36_S24_GB
DP1_F84_S24_GB
DP1_F36_S0_GB
DP2_F84_S0_GB
DM1_F12_S0_GB
DM1_F12_S24_GB
DM1_F72_S0_GB
DM1_F72_S24_GB
C1_GB
DP2_F84_S24_GB
DM2_F72_S0_GB
C2_GB
DM2_F72_S24_GB
DP2_F48_S0_GB
DP2_F48_S24_GB
DM2_F12_S0_GB
DM2_F12_S24_GB
PC2 (18 %)
PC1 (24 %)
Mannitol
Tyrosine
Lactic acid
Sucrose
Glutamic acid
Isoleucine
GABA
Malic acid
Leucine
Oxalic acid
4-CQA
Valine
5-CQA
Quinic acid
Acetic acid
Isocitric acid
Trigonelline
3-CQA
Gluconic acid
Succinic acid
4,5-diCQA
Fumaric acid
Arginine
Glutamine
Fructose
Glycerol
Threonine
Glycine
Histidine
Glucose
3,5-diCQA
3,4-diCQA
Methionine
Alanine
Lysine
Proline
Citric acid
Tryptophan
Phenylalanine
Caffeine
Aspartic acid
Asparagine
Myo-inositol
Serine
Galactose
C
Control
DM1
DM2
DP1
DP2
DP1_F84_S0
DM2_F72_S0
C2
DM1_F72_S24
DM2_F12_S0
C1
DP2_F84_S0
DM2_F72_S24
DP2_F48_S0
DM1_F12_S24
DP2_F84_S24
DP1_F36_S24
DM1_F12_S0
DM2_F12_S24
DM1_F72_S0
DP2_F48_S24
DP1_F84_S24
DP1_F36_S0
PC2 (13 %)
PC1 (35 %)
FL_BEV_Roasty
TX_BEV_Body
FL_BEV_Cereal
FL_BEV_Bitter
FL_BEV_Humus.Ea
FL_BEV_Acid
OD_BEV_Burnt
FL_BEV_Burnt
OD_BEV_Rubber
FL_BEV_Overall.intensity
OD_BEV_Malt
FL_BEV_Rubber
OD_BEV_Roasty
OD_BEV_Cocoa
OD_BEV_Spice
OD_BEV_Overall.intensity.
FL_BEV_Cocoa
OD_BEV_Humus.Earthy
FL_BEV_Spice
OD_BEV_Floral
FL_BEV_Malt
FL_BEV_Floral
OD_BEV_Cereal
TX_BEV_Astringent
OD_BEV_Fruity.
FL_BEV_Fruity.
TX_BEV_Smooth

Supplementary Figure 7. Principal component analysis of the chemical compositions of the fermenting and soaking beans (A), the chemical compositions of the green coffee beans (B), and the sensory evaluations of the brewed coffees (C) of the Arabica coffee wet processing experiments. FB, fermenting beans; SB, soaking beans.

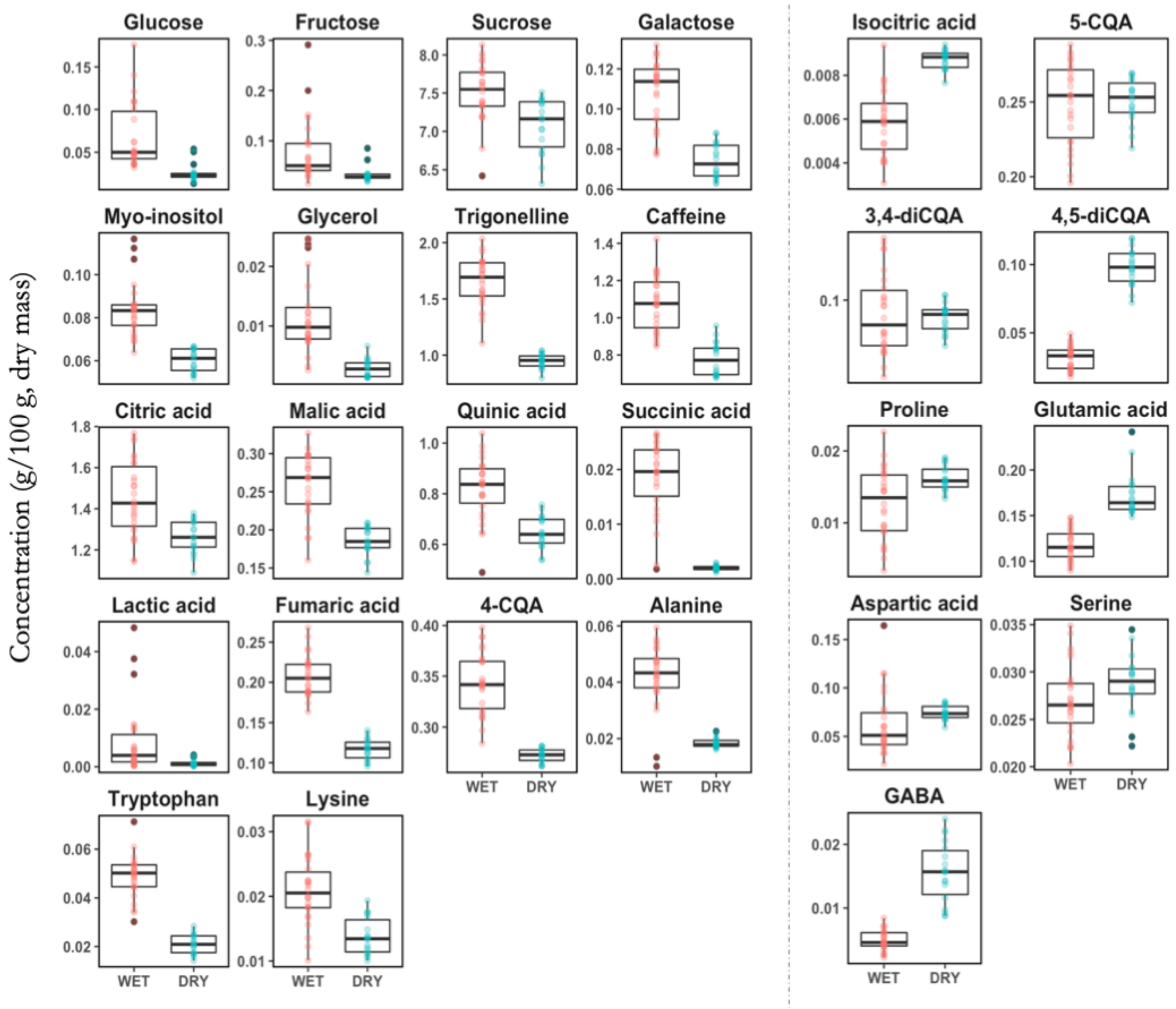

Supplementary Figure 8. Boxplots of selected metabolites of the coffee beans before and after drying of the Arabica coffee wet processing experiments. Red dots represent coffee bean samples from the fermentation and soaking steps; green dots represent the green coffee beans. CQA, caffeoylquinic acid; GABA, γ-aminobutyric acid.
